# New Insights Into the Evolution of C_4_ Photosynthesis Offered by the *Tarenaya* Cluster of Cleomaceae

**DOI:** 10.3389/fpls.2021.756505

**Published:** 2022-01-18

**Authors:** Daniele F. Parma, Marcelo G. M. V. Vaz, Priscilla Falquetto, Jéssica C. Silva, Wellington R. Clarindo, Philipp Westhoff, Robin van Velzen, Urte Schlüter, Wagner L. Araújo, M. Eric Schranz, Andreas P. M. Weber, Adriano Nunes-Nesi

**Affiliations:** ^1^Departamento de Biologia Vegetal, Universidade Federal de Viçosa, Viçosa, Brazil; ^2^Departamento de Biologia Geral, Universidade Federal de Viçosa, Viçosa, Brazil; ^3^Plant Metabolism and Metabolomics Laboratory, Cluster of Excellence on Plant Sciences, Heinrich Heine University Düsseldorf, Düsseldorf, Germany; ^4^Biosystematics Group, Wageningen University & Research, Wageningen, Netherlands; ^5^Institute of Plant Biochemistry, Cluster of Excellence on Plant Science, Heinrich Heine University Düsseldorf, Düsseldorf, Germany

**Keywords:** Cleomaceae, *Cleoserrata*, genome size, *Gynandropsis*, intermediate photosynthetic mechanism, polyploidy, *Tarenaya*

## Abstract

Cleomaceae is closely related to Brassicaceae and includes C_3_, C_3_–C_4_, and C_4_ species. Thus, this family represents an interesting system for studying the evolution of the carbon concentrating mechanism. However, inadequate genetic information on Cleomaceae limits their research applications. Here, we characterized 22 Cleomaceae accessions [3 genera (*Cleoserrata*, *Gynandropsis*, and *Tarenaya*) and 11 species] in terms of genome size; molecular phylogeny; as well as anatomical, biochemical, and photosynthetic traits. We clustered the species into seven groups based on genome size. Interestingly, despite clear differences in genome size (2C, ranging from 0.55 to 1.3 pg) in *Tarenaya* spp., this variation was not consistent with phylogenetic grouping based on the internal transcribed spacer (ITS) marker, suggesting the occurrence of multiple polyploidy events within this genus. Moreover, only *G. gynandra*, which possesses a large nuclear genome, exhibited the C_4_ metabolism. Among the C_3_-like species, we observed intra- and interspecific variation in nuclear genome size as well as in biochemical, physiological, and anatomical traits. Furthermore, the C_3_-like species had increased venation density and bundle sheath cell size, compared to C_4_ species, which likely predisposed the former lineages to C_4_ photosynthesis. Accordingly, our findings demonstrate the potential of Cleomaceae, mainly members of *Tarenaya*, in offering novel insights into the evolution of C_4_ photosynthesis.

## Introduction

C_4_ photosynthesis is a complex trait with a high degree of natural variation. It has evolved independently over 60 times in 19 different botanical families, involving anatomical and biochemical changes relative to the ancestral C_3_ state (Sage, 2004; Sage et al., 2011, 2014). Accordingly, the different origins of this photosynthetic mechanism have been tracked by means of molecular markers (Patchell et al., 2014; Bayat et al., 2018), anatomical traits (e.g., different types of Kranz anatomy; Koteyeva et al., 2011), and biochemical patterns (three types of decarboxylation enzymes; Hatch, 1988; Wang et al., 2014). In addition, the transition from C_3_ to C_4_ photosynthesis did not likely proceed through a single step, but rather *via* a series of transitory stages (Sage, 2004; Gowik and Westhoff, 2011; Schuler et al., 2016). These include the development of larger bundle sheath cells (BSCs), increase in leaf venation density (VD), restriction of glycine decarboxylase to BSCs, establishment of a photorespiratory carbon pump, enhancement of phosphoenolpyruvate carboxylase activity, establishment of the C_4_ cycle, and optimization of the C_4_ syndrome (Sage, 2004; Gowik and Westhoff, 2011). In this way, plants exhibiting C_3_–C_4_ intermediate characteristics bridge the evolutionary gap between the C_3_ and C_4_ species (Heckmann et al., 2013; Mallmann et al., 2014; Bräutigam and Gowik, 2016). Furthermore, phylogenetic studies have shown that many C_3_–C_4_ plants are closely related to C_4_ plants (McKown et al., 2005; Marshall et al., 2007; Christin et al., 2011a; Kadereit and Freitag, 2011; Patchell et al., 2014).

Cleomaceae species have been used as model to study the evolution of C_4_ photosynthesis, as this family comprises representatives of the three photosynthetic types (C_3_, C_3_–C_4_ intermediates, and C_4_) (Brown et al., 2005; Marshall et al., 2007). Additionally, Cleomaceae is closely related to Brassicaceae (sister families), which includes the C_3_ model plant *Arabidopsis thaliana*. Accordingly, the genomes of species in these families show significant synteny (Schranz and Mitchell-olds, 2006), and this close relationship has facilitated the identification of Cleomaceae orthologs in *A. thaliana.* Cleomaceae comprises nearly 200 species (Stevens, 2001; Bayat et al., 2018), three of which have been characterized as C_4_ (*Gynandropsis gynandra, Coalisina angustifolia*, and *Areocleome oxalidea*), one as a C_3_–C_4_ intermediate (*Coalisina paradoxa*), and the rest as C_3_ or C_3_-like species (Marshall et al., 2007; Voznesenskaya et al., 2007; Koteyeva et al., 2011; Bayat et al., 2018). The three Cleomaceae members characterized as C_4_ species have independent origins, as supported by phylogenetic (Patchell et al., 2014), anatomical (Koteyeva et al., 2011, 2014), and physiological evidence (Voznesenskaya et al., 2018). Therefore, this family is promising for obtaining a deeper understanding of the evolution of C_4_ photosynthesis. However, despite of that, only 15 Cleomaceae species have been thoroughly characterized as yet (Marshall et al., 2007; Voznesenskaya et al., 2007; Koteyeva et al., 2011; Bayat et al., 2018).

Furthermore, some C_3_ species of Cleomaceae deviate from the canonical C_3_ state, exhibiting variable traits associated with the C_4_ mechanism, such as increase in leaf VD, enlargement of BSCs, proliferation of both mitochondria and chloroplasts, and accumulation of transcripts and proteins required for C_4_ photosynthesis, similar to C_3_–C_4_ intermediate and C_4_ species (Marshall et al., 2007). These deviations indicate that these species may have a higher propensity for the evolution of C_4_ photosynthesis (Marshall et al., 2007), facilitating the acquisition of novel traits (Sage, 2001; Marshall et al., 2007; Mckown and Dengler, 2007; Christin et al., 2011b,2012a,b,^2013^), as observed in grasses (Christin et al., 2013). Of note, Cleomaceae species exhibiting C_3_ (*Tarenaya hassleriana*) and C_4_ (*G. gynandra*) photosynthetic metabolism share the copy number of various genes essential for C_4_ photosynthesis. However, expression analysis of C_4_ photosynthetic orthologs have demonstrated that the regulation of transcript abundance is much less stringent in *T. hassleriana* than in *G. gynandra* (Bergh et al., 2014). Thus, compared with Brassicaceae, Cleomaceae species are expected to be predisposed to the evolution of C_4_ photosynthesis, as already demonstrated for *G. gynandra* (Huang et al., 2021), given the evolutionary history of the group, including the occurrence of ancient whole genome duplications (Th-α) and paleopolyploidy events (Bergh et al., 2014).

As stated above, Cleomaceae species with C_4_ photosynthesis have independent origins. The *Coalisina* group includes C_3_, C_4_, and C_3_–C_4_ intermediate species while *Gynandropsis* and *Areocleome*, do not, since they are monotypic genera (Patchell et al., 2014; Bayat et al., 2018). In this context, additional genera/species of the family need to be characterized, as species/accessions with different photosynthetic metabolisms within the same group may facilitate the understanding of the acquisition/evolution of the C_4_ metabolism (Bayat et al., 2018). Although *G. gynandra* – the best-studied C_4_ species of Cleomaceae – does not have a close C_3_ or C_3_–C_4_ intermediate relative, studies with different accessions of this species have proven the presence of variations in the C_4_ metabolism (Reeves et al., 2018). As consequence, variations in the C_3_ and C_3_–C_4_ metabolism are also expected in other specious Cleomaceae genus, such as *Tarenaya*. Accordingly, establishment of a mapping population enables molecular marker trait association using methods such as quantitative trait locus mapping and genome-wide association studies (Reeves et al., 2018).

Despite the greatest species diversity (Stevens, 2001), few Cleomaceae species have been sampled for physiological traits to date. Brazil, for instance, is home to 34 Cleomaceae species (Bercht and Presl, 2021), most of which are endemic, but no species/accession has been physiologically characterized thus far. However, previous carbon isotope composition analysis has demonstrated the potential of this family to comprise species with the C_4_ metabolism (e.g., *T. siliculifera*, a Brazilian endemic species) (Voznesenskaya et al., 2007). Considering that Brazil has a great diversity of Cleomaceae species and presents a wide range of biomes, including (semi)arid regions, that would favor the development of species with different carbon concentrating mechanisms, we tested the hypothesis that Brazilian Cleomaceae species, sampled from different areas, are in different steps/stages of C_3_–C_4_ evolution. Beyond to this basal hypothesis, we also intended to characterize and identify the type of photosynthetic metabolism in Brazilian Cleomaceae species and, thus, gaining insights into the evolution of C_3_–C_4_ intermediates and C_4_ accessions in this family. For that, we investigated the phenotypic variability in terms of physiological (gas exchange), anatomical (diaphanization and cross section), and metabolic (sugars, starch, nitrogen compounds, and organic acids) traits. In addition, we measured the genome size of the studied species and reconstructed a molecular phylogeny based on the internal transcribed spacer (ITS) nuclear marker. In this context, we evaluated 22 phenotypically diverse accessions of Cleomaceae [distributed in 3 genera (*Cleoserrata*, *Gynandropsis*, and *Tarenaya*) and 11 species]. Accordingly, we have addressed a large number of *Tarenaya* species/accessions (the most specious Cleomaceae genus in Brazil), including species of all its series. The results are, therefore, discussed in the context of the C_3_–C_4_ and C_4_ evolutionary aspects of Cleomaceae, mainly considering *Tarenaya* species/accessions, highlighting the importance of this genus for studies on different carbon concentrating mechanisms.

## Materials and Methods

### Taxon Sampling

**FIGURE 1:**
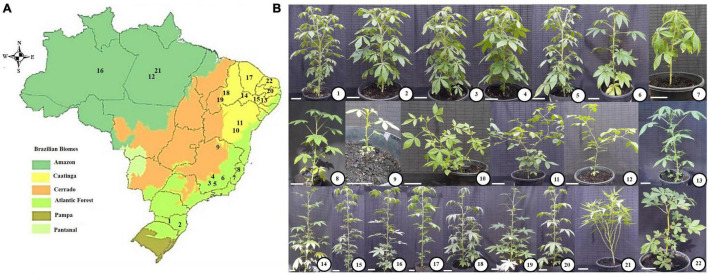
Map of Brazil with its biomes **(A)** and pictures of the studied species **(B)**. **(A)** Map showing the Brazilian biomes, in which the sampling sites of the studied species are highlighted by numbers (following the description presented in **B**). Accessions 1–8 were sampled from sites at Atlantic Forest; Accession 9 from Cerrado (Brazilian Savanna); Species 10, 11, 13–15, 17, 20, and 22 from Caatinga (Brazilian semi-arid region); Accessions 18 and 19 form transitional regions between Cerrado and Caatinga; Accessions 12, 16, and 21, from Amazon. **(B)** Pictures of the studied species, cultivated under greenhouse conditions (Please, see sections “Materials and Methods – Plant growth conditions”). **(1)** THCS: *T. hassleriana* (Canoinhas-SC). **(2)** THJ: *T. hassleriana* (Joinville-SC). **(3)** THV: *T. hassleriana* (Viçosa-MG). **(4)** THC: *T. hassleriana* (Canaã-MG). **(5)** THS: *T. hassleriana* (São Miguel-MG). **(6)** THP: *T. hassleriana* (Piau-MG). **(7)** THDM: *T. hassleriana* (Domingos Martins-ES). **(8)** TR: *T. rosea* (Colatina-ES). **(9)** TSI: *T. siliculifera* (Rio Pardo-MG). **(10)** TD: *T. diffusa* (Feira de Santana-BA). **(11)** TA: *T. aculeata* (Feira de Santana-BA). **(12)** TM: *T. microcarpa* (Belém-PA). **(13)** TIB: *T. longicarpa* (Ibimirim-PE). **(14)** TAF: *T. longicarpa* (Afrânio-PE). **(15)** TARC: *T. longicarpa* (Arcoverde-PE). **(16)** TAM: *T. longicarpa* (Manaus-AM). **(17)** TC: *T. longicarpa* (Lavras-CE). **(18)** TL: *T. longicarpa* (Picos-PI). **(19)** TS: *T. spinosa* (Teresina-PI). **(20)** TP: *T. parviflora* (Pombal-PB). **(21)** CP: *C. paludosa* (Belém-PA). **(22)** GG: *G. gynandra* (Mossoró-RN). The acronyms are followed by the species name and city/state of sampling, which are displayed between parentheses. Bars: 10 cm.

For a subset of 22 Cleomaceae species selected for the present study, comprising herbaceous and shrub species, seeds were collected *in situ* from 20 locations distributed in 11 Brazilian states (Figure 1 and Supplementary Table S1). The species used in this study were sampled from four Brazilian biomes (Amazon, Atlantic Forest, Cerrado, and Caatinga, or transitional areas between these two last) (Figure 1 and Supplementary Table S1). These biomes differ from each other mainly by the predominant climate and vegetation. Thus, the Amazon Forest has a hot and humid climate, with dense vegetation characterized by large trees. The Cerrado (Brazilian Savanna), however, has a predominantly seasonal tropical climate, with periods of rain and drought, and vegetation characterized by gnarled trees, grasses and shrubs. The Caatinga (Brazilian semi-arid region), in turn, has a semi-arid tropical climate and medium-sized shrubby vegetation, with twisted branches and leaves adapted to periods of drought. The Atlantic Forest, on the other hand, has a predominantly tropical-humid climate with high temperatures and rainfall and vegetation marked by the presence of large and medium-sized trees forming a dense and closed forest (IBGE, 2021).

After morphological identification, seven accessions were classified as *T. hassleriana* (shrub species), collected from different environments [locations: Viçosa, São Miguel do Anta, Canaã, and Piau in the state of Minas Gerais (MG); Domingos Martins in the state of Espírito Santo (ES); and Joinville and Canoinhas in the state of Santa Catarina (SC)]. Six accessions were classified as *T. longicarpa* (shrub species), collected from different environments [locations: Manaus in the state of Amazonas (AM); Ibimirim, Afrânio and Arcoverde in the state of Pernambuco (PE); Picos in the state of Piauí (PI); and Lavras in the state of Ceará (CE)]. The other species were classified as *C. paludosa* (shrub species) and *T. microcarpa* (herb species), which were collected from Belém in the state of Pará (PA); *T. aculeata* (herb species) and *T. diffusa* (herb species) collected from Feira de Santana in the state of Bahia (BA); *T. rosea* (shrub species) collected from Colatina (ES); *T. spinosa* (shrub species) collected from Teresina (PI); *T. parviflora* (shrub species) collected from Pombal in the state of Paraíba (PB); *T. siliculifera* (shrub species) collected from Rio Pardo (MG); and *G. gynandra* (herb species) collected from Mossoró in the state of Rio Grande do Norte (RN) (Figure 1 and Supplementary Table S1). The exsiccates are deposited in the VIC herbarium of the Universidade Federal de Viçosa (UFV).

The studied genera can be easily distinguished based on their morphological traits. *Cleoserrata* species are herbs with a glabrous surface, bearing leaves with three leaflets but without stipules, ebracteate inflorescences, and large smooth black seeds. *Gynandropsis* species are annual herbs with a puberulent-glandular surface, bearing leaves with three to five leaflets, ebracteate inflorescences, and small smooth black seeds. Meanwhile, *Tarenaya* species are herbs or shrubs with a puberulent-glandular or pubescent surface, bearing three to seven foliolate leaves with epinescent stipules, bracteate inflorescences, and seeds with longitudinal and transverse streaks that are not very well developed (Stevens, 2001; Bercht and Presl, 2021).

Furthermore, the different *Tarenaya* species in Brazil can also be easily distinguished from one another. *T. aculeata* bears tree foliolate leaves, small white petals, and slightly moniliform capsules; *T. diffusa* bears three to five foliolate leaves, small white petals, and fusiform capsules; *T. microcarpa* bears three to five foliolate leaves, glandular-puberulent indumentum, and small purplish petals; *T. hassleriana* bears armed petioles, glandular-puberulent indumentum, spines on the veins, and pink flowers; *T. parviflora* bears glabrescent indumentum, three to seven foliolate leaves, armed petioles, and white to deep pink petals; *T. longicarpa* bears glandular-puberulent indumentum, completely white petals or petals white at base and purple at the apex (depending on accession), and long capsules, with the fruit being two to three times longer than the gynophore (which differs from *T. spinosa*); *T. spinosa* bears glandular-puberulent indumentum and small white petals (compared with *T. longicarpa*); *T. rosea* bears glandular-puberulent indumentum and petals white at base and pink at apex; and *T. siliculifera* bears glandular-puberulent indumentum without stipular spines, small white petals, obovoid capsules (almost all species of the genus have cylindrical fruits). The different accessions of *T. hassleriana* and *T. longicarpa* could also be distinguished from one another based on height, hairiness, spine density and disposition, and inflorescence as well as flower size.

### Plant Growth Conditions

The collected seeds were germinated in 5 L plastic containers, filled with a commercial substrate supplemented with 14 g of NPK (4:14:8) per pot [0.56 g of N, 0.86 g of P_2_O_5_, and 0.93 g of K_2_O]. During spring and summer, five plants of each accession were grown in a greenhouse under semi-controlled conditions (maximum photosynthetically active radiation of 1,500 μmol photons m^–2^ s^–1^, relative air humidity of 60%, and temperature of 30 ± 2°C) and were irrigated daily. Under these conditions, the plants were grown for 2 (herbaceous species) to 5 months (shrubby species) until physiological characterization and harvesting of leaf material for anatomical and biochemical analyses.

### DNA Extraction, Amplification, and Sequencing

Total DNA was extracted from 22 fresh samples using a modified CTAB method (Doyle and Doyle, 1990). For phylogenetic analyses, the ITS regions were sequenced using the universal primers ITS 1 and ITS 4, as previously described (White et al., 1990). The fragments of interest were amplified using polymerase chain reaction (PCR). PCR products were visualized using 1% agarose gel electrophoresis, isolated, and cleaned with the Wizard SV Gel and PCR Clean-Up System (Promega). The purified fragments were then sequenced (Myleus; Belo Horizonte, Brazil). The electropherograms were visually assessed, edited, base-called, assembled, and manually aligned using Sequencer 4.1 (GeneCodes, Ann Arbor, MI, United States).

We, therefore, performed a comprehensive phylogenetic analysis by adding the ITS sequences from Cleomaceae species, recovered from the National Center for Biotechnology Information (NCBI) (Supplementary Table S2), which has shown promise for the family in previous works (see Patchell et al., 2014; Reeves et al., 2018; Soares-Neto et al., 2020). Based on these sequences, we generated a matrix with 135 taxa (648 bp in length). The results were interpretated using Bayesian inference (BI). BI analyses were performed in MrBayes using the best-fit evolutionary model, selected according to the Akaike information criterion (Posada and Buckley, 2004), with MrModeltest 2.2 (Nylander, 2004). The Markov chain Monte Carlo algorithm was executed using four runs with 50 million generations each and sampling every 5,000 generations. The first 25% trees were discarded as burn-in. The remaining trees were used to construct the majority rule consensus tree, and the posterior probability (PP) for each node was calculated.

### Flow Cytometry

Leaf fragments (∼2 cm^2^) from each Cleomaceae accession (sample) and *Solanum lycopersicum* (internal standard, 2C = 2.00 pg; Praça-Fontes et al., 2011) were co-chopped in 0.5 mL of OTTO-I nuclear extraction buffer (Otto, 1990) supplemented with 2 mM dithiothreitol and 50 μg mL^–1^ RNAse (Praça-Fontes et al., 2011). Then, 0.5 mL of the same buffer was added, and the suspensions were filtered through a 30 μm nylon mesh, placed into a microtube, and centrifuged at 100 × *g* for 5 min. The precipitate was resuspended in 100 μL of OTTO-I buffer and incubated for 10 min. The suspensions were stained with 1.5 mL of OTTO-I:OTTO-II (1:2) buffer supplemented with 2 mM dithiothreitol, 75 μg mL^–1^ propidium iodide, and 50 μg mL^–1^ RNAse (Praça-Fontes et al., 2011). The suspensions were incubated in the dark for 30 min, filtered through a 20 μm nylon mesh, and analyzed using a flow cytometer (BD Accuri C6, Accuri Cytometers, Belgium) equipped with a 488 nm laser source to promote propidium iodide emission at FL2 (615 – 670 nm) and FL3 (>670 nm). Fluorescence peaks of the G_0_/G_1_ nuclei of each Cleomaceae accession and *S. lycopersicum* were analyzed based on histograms using BD Accuri™ C6. The G_0_/G_1_ nuclear peaks exhibiting a coefficient of variation of ≤5% were considered for genome size measurement, calculated as 2*C*_*D*_ = (C1/C2) × 2*C*_*S*_, where 2*C*_*D*_ is the 2C value (pg) of each Cleomaceae accession, C1 is the mean G_0_/G_1_ peak channel of the Cleomaceae accession, C2 is the mean G_0_/G_1_ peak channel of *S. lycopersicum*, and 2*C*_*S*_ is 2.00 pg of *S. lycopersicum*. Based on previous results, the *S. lycopersicum* internal standard was replaced with *Raphanus sativus* ‘Saxa’ (2C = 1.13 pg, Praça-Fontes et al., 2011) to measure the 2C value of *G. gynandra* (GG, Mossoró-RN) and *T. siliculifera* (TSI, Rio Pardo-MG).

### Carbon Isotope Composition Analysis

Leaf material was oven-dried at 60°C for 48 h and used to determine δ^13^C and the C/N ratio. The material was analyzed using the Isoprime 100 isotope ratio mass spectrometer coupled to an isotope cube elemental analyzer (Elementar, Hanau, Germany), as described by Gowik and Bra (2011).

### Gas Exchange and Fluorescence Analyses

Fully expanded leaves from the third node of non-flowering plants were used for gas exchange and chlorophyll *a* fluorescence analysis, which were carried out with an open-flow infrared gas exchange analyzer system equipped with an integrated fluorescence chamber (IRGA, LI-COR Inc. LI-6400XT; NE). The measurements were performed during the light period between 8 and 12 h (solar time), using a 2 cm^2^ leaf chamber at 25°C, flow rate of 400 μmol CO_2_ s^–1^ and saturating light intensity of 1,000 μmol photons m^–2^ s^–1^ at the leaf level. The leaf-to-air vapor pressure deficit was maintained at 1.2–2.0 kPa, and the amount of blue light was set to 10% PPFD to optimize the stomatal aperture. Corrections for CO_2_ leakage into and water vapor from the leaf chamber of LI-6400 were applied to all gas exchange data, as previously described (Rodeghiero et al., 2007). Dark respiration (*R*_d_) was measured using the same gas exchange system described above, on the same leaves used for determination of the gas exchange parameters, after at least 1 h of acclimation in the dark (Rodeghiero et al., 2007).

### Metabolite Analyses

Fully expanded leaves from the third node of non-flowering plants were harvested in the middle of the light period, soon after gas exchange and fluorescence analyses. At this point, the herbaceous plants were 2-month-old and the shrub species were 5-month-old. The samples were snap-frozen and stored at –80°C until analysis. Metabolites were extracted by grinding the material in liquid nitrogen and adding the appropriate extraction buffers. Approximately 25 mg of the freeze-dried leaf matter was subjected to ethanolic extraction at 70°C for 30 min. After centrifugation (at 21,800 × *g* for 5 min), the total chlorophyll content (*a* + *b*) and chlorophyll *a*/*b* ratio were determined as previously described (Porra et al., 1989).

The concentration of glucose, fructose, and sucrose was determined from the liquid phase, as described previously (Fernie et al., 2001). Total free amino acid (Cross et al., 2006), malate, and fumarate (Nunes-Nesi et al., 2007) contents were determined as previously described. From the ethanol insoluble fraction, the concentration of starch (Fernie et al., 2001) and total soluble proteins (Bradford, 1976) was determined according to standard protocols.

### Metabolite Profiling

Fully expanded source leaves from each sampled plant were collected in the middle of the light period, wrapped in aluminum foil, flash frozen in liquid nitrogen, and stored at –80°C until analysis. After lyophilization and grinding, approximately 15 mg dry weight was extracted for gas chromatography–mass spectrometry (GC–MS) in 1.5 mL of extraction solution (water:methanol:chloroform, 1:2.5:1 *v/v*) as described by Fiehn (2007), using 5 μM ribitol (Sigma-Aldrich) as the internal standard. The extract (15 μL) was dried in a vacuum concentrator and derivatized in two steps using the MPS Dual Head autosampler (Gerstel), as described by Gu et al. (2012). After incubation for 2 h at room temperature, the samples were analyzed as described previously (Shim et al., 2020) using the 5977B GC-MSD system (Agilent Technologies). The peaks were integrated using MassHunter Quantitative (v b08.00; Agilent Technologies). For relative quantification, metabolite peak areas were normalized to the corresponding dry weight and peak area of the internal standard.

### Micromorphological Analyses

Anatomical analysis was conducted to evaluate the VD of fully developed leaves in the upper third. Diaphanization of the plant material was performed as described previously (Zsögön et al., 2015). Subsequently, the samples were assembled on glass slides, and images of the adaxial epidermis were obtained using the Zeiss Axio Scope A1 photomicroscope coupled to a color image capture system (Axiovision ^®^ 105). Five photographs of each slide were obtained to cover the entire leaf length. In addition, five replicates (five leaves per individual) were collected and analyzed. Vein density was calculated as the length of the second-, third-, and fourth-order veins in a given leaf area (mm mm^–2^). Quantification was performed using Image Pro-Plus ^®^ (v 4.5; Media Cybernetics, Silver Spring, MD, United States). In addition, the median region of the fully expanded leaves from the third node was collected and fixed in formaldehyde:acetic acid:ethanol 50% (FAA50) at a ratio of 5:5:9 (*v/v*) for 48 h. The material was dehydrated in an ethanolic series and embedded in historesin (Leica, Heidelberg, Germany). Transverse sections (thickness, 5 μm) were obtained using a self-advancing rotary microtome (RM 2155, Leica, Heidelberg, Germany) with glass razors. The sections were stained with 0.05% toluidine blue in acetate buffer (pH 4.7) (O’Brien et al., 1964) for 1 min and assembled between slides and coverslips with synthetic resin. Photomicrographs were obtained using a photomicroscope (Zeiss, MC-80). The micrometric scales were photographed and enlarged under the same optical conditions. Leaf thickness was measured considering the entire length of the epidermis. For each slide analyzed, five measurements were obtained in different regions to cover the entire area. The distance between the vascular bundles was measured between the sides of the veins. Bundle sheath cell width (BSCW) was measured radially in five cells at random on each slide (cells from different vascular bundles were selected when possible). Leaf thickness, palisade parenchyma, spongy parenchyma, intervein distance, and BSCW were measured (Supplementary Figure S1) using Image Pro-Plus ^®^.

### Statistical Analyses

Data were obtained from five plants per accession, each placed in an individual pot; thus, each pot represented a biological replicate. All plants were completely randomized. The effect of accession was determined using analysis of variance (*P* < 0.05). Means were analyzed using Tukey’s test.

Phylogenetic generalized least squares (PGLS) regression was applied. We used a phylogenetic tree constructed using BI based on the ITS sequences of 21 novel Brazilian accessions (Supplementary Figure S2) and a matrix of 74 variables. The species *G. gynandra* (C_4_ species) was removed from the analysis, as its genome size values (2–4 times greater compared to the other species in this study) and results obtained from other analyzes could bias/influence the regression result. The script available at https://lukejharmon.github.io/ilhabela/instruction/2015/07/03/PGLS/ was used. PGLS uses the common statistical mechanism of generalized least squares and applies it to phylogenetic comparative data (Felsenstein, 1985a). This method allows for the estimation of the impact of phylogeny on the covariance among residuals, thereby controlling for relatedness. In the case of Brownian motion, residues with variances and covariances follow the structure of the phylogenetic tree (Felsenstein, 1985b). All statistical analyses were performed using Statistica and R.

## Results

The Cleomaceae species selected for this study were sampled from 20 sites, which were found in four Brazilian biomes (Amazon, Atlantic Forest, Cerrado, and Caatinga) (Figure 1 and Supplementary Table S1). In this sense, the biomes with the highest number of sampled specimens were the Atlantic Forest (11 spp.) and Caatinga (7 spp.). However, beyond the differences in climate and vegetation described above, these biomes also show great diversity considering its phytophysiognomies. Accordingly, the Atlantic Forest has 12 ecosystems/phytophysiognomies (IBGE, 2021) while Caatinga has six main phytophysiognomies (Andrade-Lima, 1981). Interestingly, it was also possible to observe that accessions of the same species (mainly *Tarenaya hassleriana* and *T. longicarpa*), collected from different sites showed phenotypic/morphological plasticity (Figure 1). More important, as described below, in addition to the phenotypic plasticity, accessions from different locations also displayed great genetic and physiological diversity.

### Genome Size in Cleomaceae

**FIGURE 2:**
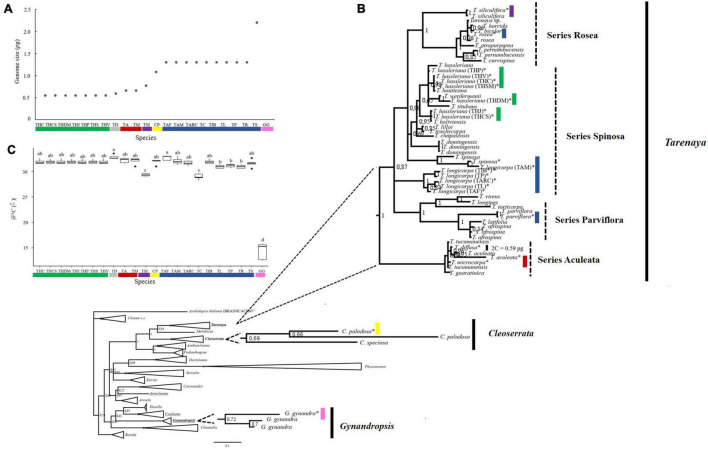
Genome size, carbon isotopic composition and molecular phylogeny of Cleomaceae species. **(A)** Genome size. **(B)** Bayesian Inference consensus tree inferred from nuclear ribosomal ITS sequences retrieved from Cleomaceae and Brassicaceae members, demonstrating the phylogenetic relationship between Cleomaceae genera. Underlined sequences represent genera that have species with C_4_ photosynthetic metabolism (*Gynandropsis, Coalisina*, and *Areocleome*). In bold are the genera analyzed in this study (*Tarenaya*, *Cleoserrata*, and *Gynandropsis*). The sequences generated in this study are marked with an asterisk. In addition, the 2C nuclear value for each species (group) was added following its identification. The colors represent different genome sizes, as described in **(A)**. Numbers at nodes reflect PP. Bar: 0.2 nucleotide substitutions per site. **(C)** Carbon isotope composition (δ^13^C; ^•^_/••_). Letters above individual box-scatter indicate significant groupings according to Tukey’s Test (*n* = 5). The median is indicated by solid lines in each box; data dispersion is represented by the interquartile range, followed by standard error and outliers. Species studied: The acronyms are those described in Figure 1.

In flow cytometry histograms with G_0_/G_1_ peaks of nuclear suspensions, the coefficient of variation for all Cleomaceae species/accession and the internal standards (*S. lycopersicum* or *R. sativus*) was < 3.70%. Based on genome size, the 22 accessions were separated into seven groups: (i) *T. hassleriana* accessions (THC, THCS, THDM, THJ, THP, THS, and THV) with 2C = 0.55 pg ± 0.003; (ii) *T. diffusa* (TD) with 2C = 0.59 pg ± 0.004; (iii) *T. aculeata* and *T. microcarpa* (TA and TM) with 2C = 0.66 pg ± 0.004; (iv) *T. siliculifera* (TSI) with 2C = 0.77 pg ± 0.003; (v) *C. paludosa* (CP) with 2C = 1.08 pg ± 0.004; (vi) nine *Tarenaya* species (TS, TP, TL, TR, TAF, TAM, TARC, TC, and TIB) with 2C = 1.30 pg ± 0.014; and (vii) *G. gynandra* (GG) with 2C = 2.20 pg ± 0.002 (Figure 2A). This pattern found for genome size was somehow unexpected, mainly because most species belong to the same genus. Accordingly, indicating the high degree of variation, mainly of *Tarenaya* species/accessions, which are spread into five different groups. Thus, we observed intrageneric and interspecific variation in nuclear genome size among the Brazilian Cleomaceae species. Based on the mean 2C values and phylogeny (Figure 2B), we speculate that increase and decrease in genome size contributed to the diversification of Cleomaceae species, specifically the C_4_ species *G. gynandra* (2C = 2.20 pg, fourfold higher than that of *T. hassleriana*). Considering the correlation between the 2C value and the 2n chromosome number of *Tarenaya* species and *G. gynandra*, we hypothesize that euploidy and disploidy (ascendant and/or descendant) resulted in interspecific variations in the 2C value and C_4_ photosynthetic mechanism of *G. gynandra*. Alternatively, genome size differences may be attributable to whole genome duplications (Mabry et al., 2020) and/or changes in repetitive DNA content (Beric et al., 2020, 2021).

### Phylogeny of Cleomaceae Accessions

Our Bayesian phylogenetic reconstruction based on the ITS sequences of Cleomaceae species indicated that most of our analyzed accessions were positioned in the *Tarenaya* genus. The exceptions were the sequences of *C. paludosa* and *G. gynandra*, which were placed into the *Cleoserrata* and *Gynandropsis* clusters, respectively (Figure 2B). Moreover, *Tarenaya* is sister to two American genera, namely *Cleoserrata* and *Melidiscus*, whereas *Gynandropsis* is sister to the African cluster comprising *Coalisina* and *Iltisella* as well as to *Cleomella* (Figure 2B). These results are somehow contrary to the findings reported by Bayat et al. (2018), who used five molecular markers (ITS, *mat*K, *ndh*F, *ycF*, and rps) and another set of species/sequences. Interestingly, *Gynandropsis*, *Areocleome*, and *Coalisina*, which harbor species with C_4_ photosynthetic metabolism, formed different clusters (Figure 2B), reinforcing that this photosynthetic mechanism has evolved independently at least three times within the family, as was previously reported (Patchell et al., 2014; Bayat et al., 2018).

The analyzed genera, namely *Gynandropsis*, *Cleoserrata*, and *Tarenaya* are monophyletic. Within the *Tarenaya* group, the series Rosea, Parviflorae, and Aculeatae are monophyletic; the Spinosa series, which includes the type species of the genus (*T. spinosa*), is polyphyletic; and the Aculeatae series, which includes herbaceous species bearing small flowers, is placed more externally to the other series of the genus (Figure 2B). Within the Spinosa series, *T. hassleriana* accessions form different sub-clusters, indicating high genetic diversity among the species (Figure 2B). Overall, the 20 accessions of *Tarenaya* studied here cover a great genetic diversity into this genus, mainly considering that species from all series were sampled and were represented in all analysis.

### Carbon Isotope Composition

The carbon isotope composition of plants has been widely used to identify the photosynthetic mechanisms, and it can, therefore, assist phylogenetic studies exploring the evolution of C_4_ photosynthesis (Von Caemmerer et al., 2014; Yang et al., 2017). Hence, to help identifying the photosynthetic mechanism of the accessions studied here and to merge these data with molecular phylogeny, we performed carbon isotope composition (δ^13^C) analysis using fully expanded leaves of plants growing under the same conditions. In the Cleomaceae accessions tested, the δ^13^C value ranged from –15‰ to –32‰, representing at least two types of photosynthetic mechanisms, namely C_3_ and C_4_ (Figure 2C). As expected, the Brazilian accession of *G. gynandra* exhibited the highest δ^13^C value (–15‰), and the other species showed values around –32‰. Meanwhile, *T*. *siliculifera* and *T*. *longicarpa* (Lavras, CE) exhibited δ^13^C values close to –29‰, being significantly different from the other accessions (Figure 2C), highlighting a certain degree of variation among the C_3_-like species found into the genus *Tarenaya*.

### Phylogenetic Generalized Least Squares Regression

In order to determine the correlations of genome size with the other study variables, independently, in the phylogenetic topology of the Brazilian Cleomaceae accessions, we decided to perform PGLS regression analysis (Table 1 and Supplementary Figures S3, S4). As the size of the *G. gynandra* genome is 2–4 times greater compared to the other species in this study, we removed this species from the analysis to avoid bias/influence the regression result.

**TABLE 1:** Variables that were significant (*p* < 0.05) in PGLS model with genome size.

			Minimum	Mean	Maximum	*SE*	CV	Correlation	*P*
**Physiological parameters**		*A*_N_ (μmol CO_2_ m^–2^s^–1^)	7.31	19.56	31.86	0.73	0.17	+	0.003
		*R*_d_ (μmol CO_2_ m^–2^s^–1^)	0.70	1.37	1.80	0.06	0.21	+	0.01
		WUEi (μmol m^–2^ s^–1^)	29.65	44.15	77.59	2.57	0.27	–	0.001
		A_gross_ (μmol CO_2_ m^–2^s^–1^)	14.85	27.52	36.70	0.68	0.11	+	0.001
**Biochemical parameters**		| δ^13^*C* (^•^_/••_)|	28.97	31.56	32.79	0.14	0.02	–	0.001
		Proteins (μmolg–^1^ DW)	6.63	8.91	13.53	0.28	0.15	–	0.001
		Starch (μmol glucose g^–1^ DW)	0.01	0.02	0.06	0.00	0.42	+	0.001
		Chlorophyll *a* + *b* (μmol g^–1^ DW)	5.95	29.89	156.08	4.19	0.64	+	0.001
**Anatomical parameters**		Palisadic parenchyma (μm)	7.76	14.23	17.58	0.48	0.15	+	0.001
		Density of venation (mm mm^–2^)	15.95	24.99	48.79	1.17	0.22		0.018
**Metabolites data**	**Amino acids**	α-Alanine	251.13	472.96	2226.79	41.53	0.40	+	0.001
		Glutamine	0.04	0.06	0.11	0.00	0.13	+	0.001
		Glycine	9.26	32.97	116.09	3.68	0.51	+	0.001
		Leucine	5.14	45.59	110.89	6.32	0.64	+	0.001
		Ornithine	0.05	0.07	0.12	0.00	0.13	+	0.042
		Phenylalanine	54.55	174.23	541.60	17.05	0.45	+	0.031
		Proline	55.46	130.42	210.29	7.70	0.27	–	0.001
		Methionine	72.58	160.90	311.50	10.37	0.30	+	0.001
		Putrescine	36.67	90.91	384.39	8.88	0.45	+	0.001
		Serine	0.08	0.14	0.23	0.00	0.12	+	0.003
		Tyrosine	492.03	1480.16	6638.51	165.25	0.51	+	0.01
	**Organic acids**	Aspartate	3.36	21.66	93.91	2.98	0.63	+	0.001
		GABA	236.29	382.87	1116.21	27.38	0.33	+	0.005
		Gluconate	17.61	54.62	229.72	4.85	0.41	+	0.001
		Glycerol	16.80	53.39	114.24	4.94	0.42	+	0.001
		Isocitrate	1239.68	7818.80	37737.81	743.17	0.44	+	0.036
		Malate	6.39	27.32	93.11	3.11	0.52	+	0.002
		Malonate	431.45	1712.42	3809.49	157.24	0.42	–	0.003
	**Sugar**	Glucose	15.32	104.02	1013.09	20.35	0.90	+	0.001
		Myo-Inositol	6.06	164.93	1257.78	31.70	0.88	+	0.003
**Development**		Specific leaf area (m^2^ mg^–1^)	249.45	371.57	503.55	18.26	0.23	–	0.001
		Weight 1000 seeds (g)	1.06	2.16	3.07	0.09	0.20	–	0.001

*The raw data are plotted with the phylogenetic generalized least squares regression line in Supplementary Figures S2, S3.*

It is noteworthy that a phylogeny, based on the studied species (except *G. gynandra*), was built exclusively for PGLS. Thus, with a smaller sample of species and a single molecular marker (ITS), it is possible to see that the *Tarenaya* series were no longer monophyletic (Supplementary Figure S2). PGLS analysis revealed that 32 out of 74 variables analyzed here were significantly correlated to genome size (*P* < 0.05) (Table 1). Based on the significant regressions, the models with gas exchange parameters (*A*_N_, *A*_gross_ and *R*_D_), leaf anatomical traits (size of palisade parenchyma and VD), organic acids (malate, isocitrate, and gluconate), amino acids (aspartate, α-alanine, methionine, phenylalanine, tyrosine, glutamine, serine, glycine, and leucine), other nitrogen-metabolism related compounds (γ-aminobutyric acid, GABA), putrescine and ornithine), total clorophyll, glucose, and glycerol were positively correlated with genome size. Interestingly, the δ^13^C value, intrinsic water use efficiency (WUEi), malonate, myo-inositol, proline, starch content, proteins content, seeds weight and specific leaf area were negatively correlated with genome size (Table 1 and Supplementary Figures S3, S4).

### Physiological Parameters

**FIGURE 3:**
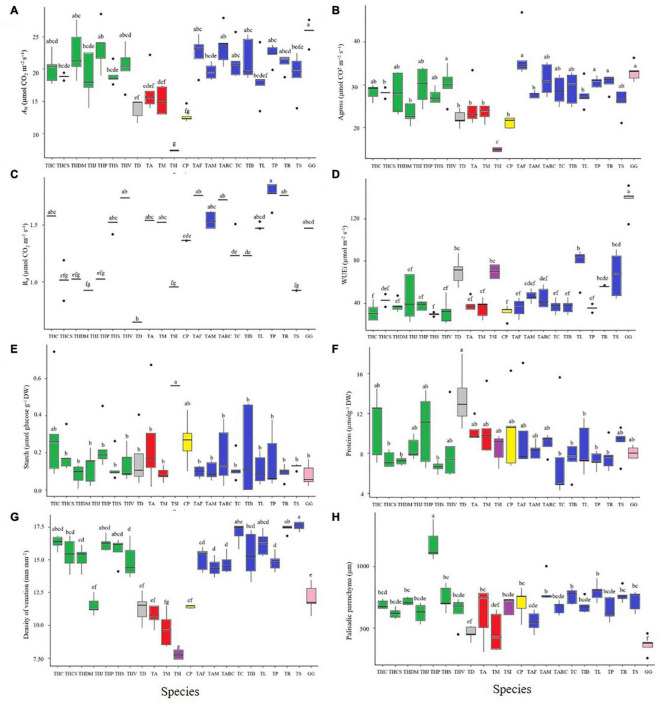
Gas exchange and chlorophyll *a* fluorescence parameters in Cleomaceae species. Physiological variables: **(A)** Ambient CO_2_ assimilation rates (*A*_N_) (400 ppm atmospheric [CO_2_]). **(B)** Stomatal conductance (*g*_s_). **(C)** Dark respiration (*R*_d_). **(D)** Intrinsic water use efficiency (WUEi). Metabolic variables: **(E)** Starch content. **(F)** Protein content. Anatomical variable: **(G)** Density of venation. **(H)** Palisade parenchyma. Letters above individual box-scatter indicate significant groupings according to Tukey’s Test (*n* = 5). The median is indicated by solid lines in each box; data dispersion is represented by the interquartile range, followed by standard error and outliers. The colored bars as well as the species acronyms are related to the groups observed in Figures 1, 2.

To completely characterize and understand the physiological basis for the differences among the studied Cleomaceae accessions, we performed detailed physiological analyses of diffusional, photochemical, and biochemical constraints on photosynthesis (Figures 3A–D and Supplementary Figure S5). Under the tested environmental conditions (as described in the section “Materials and Methods”), Cleomaceae accessions showed natural variation, even within the same species, in terms of physiological parameters related to photosynthesis. In general, the studied Cleomaceae accessions exhibited a high *A*_N_, with values greater than 20 μmol CO_2_ m^–2^⋅s^–1^ (Figure 3A and Table 2). However, the herbaceous accessions of *T. aculeata*, *T. diffusa*, and *T. microcarpa* exhibited the *A*_N_ of ∼10 μmol CO_2_ m^–2^⋅s^–1^. While the shrubby accession of *T. siliculifera* showed the *A*_N_ of <10 μmol CO_2_ m^–2^⋅s^–1^. *T. siliculifera* exhibited the lowest stomatal conductance (*g*_s_), followed by *G. gynandra*, herbaceous species (*T. aculeata*, *T. diffusa*, and *T. microcarpa*), and *Tarenaya* species (Spinosa I and II, Parviflorae, and Cleorosea) (Supplementary Figure S5). For the internal CO_2_ concentration (*C*_i_), which is related to *g*_s_, the same pattern as that for *g*_s_ was observed (Supplementary Figure S5). Regarding transpiration (*E*) and *R*_d_, the studied Cleomaceae accessions exhibited substantial differences with no clear pattern between the groups (Figure 3 and Supplementary Figure S5). Similarly, WUE_i_ was remarkably higher in *G. gynandra*, albeit without a clear trend in the rest of the species (Figure 3).

**TABLE 2:** Physiological variation for photosynthetic gas-exchange and anatomical parameters among diverse species of Cleomaceae, Brassicaceae, and Asteraceae.

Species	*A*_N_ (μmol m^–2^s^–1^)	δ^13^C (^•^_/••_)	Vein density (mm mm^–2^)	References
*Gynandropsis gynandra* – C_4_	23–35	–14 to 17	6–10	Marshall et al., 2007; Voznesenskaya et al., 2007; Koteyeva et al., 2011; Reeves et al., 2018
Cleomaceae	28	–15	11–12.5	Present study

*Tarenaya hassleriana* – C_3_	–	–24 to 30	–	Marshall et al., 2007; Voznesenskaya et al., 2007
Cleomaceae	18–24	–32	12–16	Present study

*Tarenaya spinosa* – C_3_	–	–26 to 30	5	Marshall et al., 2007; Voznesenskaya et al., 2007
Cleomaceae	20	–31	17	Present study

*Coalisina paradoxa* – C_3_– C_4_	–	–	9–11	Marshall et al., 2007
Cleomaceae	18	–26	–	Voznesenskaya et al., 2007

*Arabidopsis thaliana –* C_3_	16	–	1.5–3	Stewart et al., 2018
Brassicaceae	10–16	–27 to 30	–	Easlon et al., 2014

*Moricandia moricandioides –* C_3_ Brassicaceae	28	–31 to 36	4–6	Schlüter and Weber, 2016
*Moricandia suffruticosa –* C_3_– C_4_ Brassicaceae	25	–31 to –33	3.5–4.5	
*Moricandia arvensis –* C_3_– C_4_ Brassicaceae	25	–30 to 37	4–6	

*Flaveria robusta* – C_3_ Asteraceae	–	–	2.65	McKown and Dengler, 2009
*Flaveria bidetins* – C_4_ Asteraceae	–	–	2.89	

### Biochemical Analyses

To unveil the natural variations among the different Cleomaceae accessions studied, we performed detailed metabolite analyses using leaf samples. First, we examined the levels of the major carbon- and nitrogen-containing metabolites in leaves harvested at the middle of the light period. Overall, despite clear variations in physiological traits (Figures 3E,F and Supplementary Figure S6), the steady-state levels of soluble sugars (glucose, fructose, and sucrose), starch, amino acids, proteins, and chlorophylls did not differ among the plants of different species (Figures 3E,F and Supplementary Figure S6). The only exception to this trend was *T. siliculifera*, which exhibited higher steady-state levels of sugars and starch than the other species (Figure 3E and Supplementary Figure S6). Meanwhile, *G. gynandra* exhibited higher levels of amino acids (Figure 3E) and proteins than *T. diffusa* (Figure 3F). There were no significant differences in the chlorophyll *a*/*b* ratio among the studied species (Supplementary Figure S6).

Furthermore, we performed metabolite profiling of leaf samples from all studied accessions using GC–MS. This analysis allowed for the identification and quantification of 33 metabolites (Supplementary Table S3 and Supplementary Figure S7). For 19 metabolites analyzed, we did not observe significant differences among the studied accessions. Among these metabolites, there were no differences in the levels of the amino acids aspartate, glycine, and serine or the organic acids citrate and isocitrate. In contrast, *T. siliculifera* exhibited significantly higher levels of myo-inositol, succinate, malonate, malate, threonate, glycerate, glycerol, glucose, fructose, and sucrose. In addition, *T. hassleriana* exhibited higher levels of methionine, glutamate, and alanine.

### Leaf Anatomy

In leaves, the formation of venation, plasmodesmata, and other barriers to gas diffusion is important for photosynthetic efficiency. Thus, the variability in leaf anatomical traits, which are related to the carbon concentrating mechanism, was determined in the Cleomaceae accessions studied. Leaf anatomical parameters were evaluated by diaphanization and transverse sectioning to determine VD and BSCW. *T. hassleriana* (THC, THCS, THP, and THS), *T. longicarpa* (TP, TC, and TIB), *T. rosea*, and *T. spinosa* showed higher VD than the other analyzed accessions (Figure 3G). Meanwhile, *T. hassleriana* (THDM and THV), *T. longicarpa* (TAF, TAM, and TARC), and *T. parviflora* showed intermediate VD, whereas *G. gynandra*, *T. hassleriana* (THJ), *T. diffusa*, *T. aculeata*, *T. microcarpa*, *T. siliculifera*, and *C. paludosa* exhibited lower VD (Figure 3G).

Bundle sheath cell width was higher in *G. gynandra*, *T. hassleriana* (THC and THCS), *T. siliculifera*, and *T. longicarpa* (TAM) than in the other accessions studied (Supplementary Figure S8). Meanwhile, *T. diffusa, T. aculeata*, *T. microcarpa*, *T. parviflora*, and *T. longicarpa* (TARC and TAF) exhibited lower BSCW. The remaining 11 accessions exhibited intermediate BSCW (Supplementary Figure S8).

The interveinal distance (ID) was higher in *T. microcarpa* but lower in *G. gynandra* and *T. siliculifera*. The other accessions showed intermediate ID (Supplementary Figure S8). *T. hassleriana* (THP) showed the highest leaf thickness, whereas *T. diffusa* and *T. microcarpa* showed the lowest leaf thickness. The other accessions exhibited intermediate values and did not differ in terms of thickness (Supplementary Figure S8). Regarding the thickness of the palisade (Figure 3H) and spongy parenchyma (Supplementary Figure S8), *T. hassleriana* (THP) exhibited higher values and *G. gynandra* showed lower values than the remaining accessions, which showed intermediate values (Supplementary Figure S8).

Surprisingly, we could not separate the accessions into groups based on these morphoanatomical characteristics, which would be expected considering that the C_4_ species *G. gynandra* could be clearly distinguished from the other C_3_ species in terms of most of the analyzed parameters (e.g., VD and BSCW) (Supplementary Figures S9, S10). In this context, our results suggest that the analyzed accessions diversified at the species level. Since *T. hassleriana* and *T. longicarpa* differed in terms of the analyzed parameters, anatomy would provide additional evidence of this diversification. Although these traits indicate the possible diversification in photosynthetic mechanisms, physiological and biochemical data provide additional evidence.

## Discussion

### Interspecific Variations in the 2C Value Complement the Molecular Phylogeny of Cleomaceae

Cleomaceae is a highly diverse family, both morphologically (e.g., monoecious, dioecious, polygamous species; e.g., Omondi et al., 2017; Zohoungbogbo et al., 2018; Riaz et al., 2019) and physiologically (species with different photosynthetic metabolisms: C_3_, C_3_–C_4_, and C_4_). This diversity was represented by species studied here, which were morphologically (*Tarenaya*, *Cleoserrata*, and *Gynandropsis*; Stevens, 2001; Bercht and Presl, 2021) and physiologically (e.g., *Tarenaya* and *Gynandropsis*; Marshall et al., 2007; Bayat et al., 2018) distinct. The three genera analyzed here, namely *Tarenaya*, *Cleoserrata*, and *Gynandropsis*, were monophyletic (Figure 2). Of note, however, *Gynandropsis* is a monotypic genus (*G. gynandra*). *Tarenaya*, one of the most diverse genera in terms of the number of species within Cleomaceae, also includes many morphologically distinct species, which allows for the separation of the genus into series (small groups of species) (e.g., Iltis, 1957, 1959; Hall et al., 2002; Sanchez-Acebo, 2005; Inda et al., 2008; Feodorova et al., 2010; Iltis and Cochrane, 2014). These series have previously been shown to be monophyletic (see Soares-Neto et al., 2020), and our results are largely congruent with this report, with the exception of the Spinosa series, which we found to be polyphyletic. Of note, our study is limited by the number of samples of this rather diverse series (see Soares-Neto et al., 2020). Thus, studies with increased taxon sampling or markers are warranted to resolve the monophyly of the Spinosa series.

The morphological and physiological diversity can be linked to variations in genome size, among other factors (Paterson et al., 2010; Roddy et al., 2019). Thus, an interspecific variation at the diploid level, for example, as found for the species *T. hassleriana, T. diffusa, T. aculeata*, and *T. microcarpa*, may suggest that the DNA content may be a parameter that can be used to differentiate the species (e.g., *Miscanthus* 2C = 3.9 to 6.9 pg – Sheng et al., 2016). However, the observed species and genome size diversity was not consistent with the molecular phylogenetic results (Figure 2; Patchell et al., 2014; Bayat et al., 2018). This pattern is different, for example, from that observed for *Lupinus* (Fabaceae). In this genus, the genome size (0.97–2.44 pg) data supports the generally accepted taxonomic classification of the Old World lupins (Naganowska et al., 2003). In this sense, considering members of Cleomaceae, we observed that different series have species with the same genome size (Parviflora, Rosea, and Spinosa). Furthermore, within the same series it was also possible to observe representatives with different genome sizes (e.g., *T. hassleriana* and *T. spinosa* – Spinosa series, and *T. rosea* and *T. siliculifera* – Rosea series) (Figure 2). It is noteworthy that the studied Cleomaceae species have an average genome size of 0.5 – 2.2 pg, which is larger than that recorded for *Arabidopsis*. However, in line to most species that have their genome quantified (Bai et al., 2012), such as rice, tomatoes and fruit–cherry, mango, papaya, orange, and peach (Arumuganathan and Earle, 1991).

Based on the variations in genome size among Cleomaceae genera and even among species of the same genus (e.g., *Tarenaya*; Figure 2A), even our small sample of the family reflects the relatively large interspecific variability. The intraspecific variations in the ITS sequence as well as the interspecific variations in this sequence and the 2C value may be related to different selective environmental pressures. Polyploidy, disploidy (ascendant and/or descendant), and genome size are associated with life-history traits, including vegetative form, flowering time, and adaptation to particular ecological niches, generating a basis for evolutionary novelty (Gregory, 2002; Chris Pires et al., 2006; Defoort et al., 2019). It is noteworthy that the species studied here have different forms of life (herbaceous *vs.* shrubby) and occur in different environments/biomes and consequently occupy different niches (Figure 1 and Supplementary Table S1). Nonetheless, only a few Cleomaceae species have been characterized in terms of their chromosome number (Ammal, 1933; Subramania and Susheela, 1988; Inda et al., 2008) and/or genome size (Omondi et al., 2017), and further efforts are required to understand the karyotype evolution in these taxa.

The events of gene/genome duplication observed for members of Cleomaceae contributed to several aspects of the evolution of C_4_ photosynthesis in *G. gynandra* (Huang et al., 2021). Accordingly, this species has retained the duplicates of alanine aminotransferase and glutamine oxoglutarate aminotransferase. Besides almost all known vein-development-related paralogous genes derived from the genome duplication, this event also facilitated the evolution of C_4_ enzyme genes and their recruitment into the C_4_ pathway. In addition, several genes encoding photosystem I proteins were derived from the genome duplication (for more details see Huang et al., 2021). Furthermore, there are some examples of the duplication of gene copy number and its outcomes in the evolution of C_4_ metabolism, such as the phosphoenolpyruvate carboxylase kinase gene families and the carbonic anhydrase genes (Wang et al., 2009). However, the number of enzymes required for C_4_ metabolism is limited. In addition, the duplicated genes and other genomic regions often produce different effects (Lynch and Conery, 2003) and do not always result in functional innovation (Nielsen et al., 2005). In this context, some studies have proposed the involvement of parallel evolution in changes of gene expression and amino acid sequences (Christin et al., 2012a,b; Williams et al., 2012). Therefore, the *cis*-elements that direct cell specificity in C_4_ leaves are present in C_3_ orthologous genes recruited to the C_4_ pathway, probably facilitating parallel evolution (Williams et al., 2012).

### The 2C Value and Characteristics of Carbon Concentrating Mechanisms in Cleomaceae

The increase and/or decrease in DNA content can promote phenotypic changes in the biochemical, anatomical, and physiological aspects of photosynthesis (Warner and Edwards, 1993; Vyas et al., 2007; Oa et al., 2011; Roddy et al., 2020). The importance of species relationships in trait-based studies is well appreciated in the field of comparative biology (Guy et al., 2019). Accordingly, *G. gynandra*, the species with C_4_ photosynthesis, possesses a larger genome (Figure 2A). Nonetheless, a critical component in the study of adaptations is the identification of evolutionary correlations between phenotypic characteristics in phylogenetically close species (Adams, 2014). In this context, phylogenetic regression analysis provides a flexible analytical tool for assessing the degree of evolutionary association between variables while accounting for phylogeny (Adams, 2014).

The species with intermediate genome size (2C = 1.30 pg) (Figure 2A) had shorter ID and BSCW values, similar to the C_4_ species *G. gynandra* (Figure 3 and Supplementary Figure S8). The lowest ID observed in *G. gynandra* can be attributed to the decline in the mesophyll (M) cell number, as verified, for instance, by Marshall et al. (2007) and Reeves et al. (2018), and size, which are considered essential for optimal C_4_ function, since fewer M cells reduce the mean diffusion distance for C_4_ metabolites (Hattersley, 1984; Khoshravesh et al., 2020). In addition, the enlargement of BSCs (equivalent to that in C_4_ plants) is considered essential for C_4_ function; as such, larger BSCs allow for a greater organelle volume and create larger vacuoles, serving as a barrier to facilitate CO_2_ trapping in the sheath tissues (Von Caemmerer and Furbank, 2003; Khoshravesh et al., 2020). Consistent with previous reports, our results (Figure 2A) indicated that increase in the genome size affected leaf morphological and anatomical traits, in addition to physiological function (Roddy et al., 2020). Although the mechanism underlying the effect of genome size on cellular morphology remains unknown (Roddy et al., 2020), its effects on the vascular transport network, vessel size, and density have already been demonstrated (Maherali et al., 2009; Hao et al., 2013; De Baerdemaeker et al., 2018; Simonin and Roddy, 2018). In agreement, it has been suggested that genome size is a better predictor of guard cell size and stomatal density (Simonin and Roddy, 2018). Therefore, genome size can act as a first-order restriction on carbon gain, which directly affects the upper limit of allocation for growth, reproduction, and defense (Roddy et al., 2020).

Based on this premise, genome size may affect the tolerance of environmental changes related to latitude, altitude, temperature, precipitation, salinity, and desiccation (Grime and Mowforth, 1982; Otto and Whitton, 2000; Díez et al., 2013; Wang et al., 2018; Silva Artur et al., 2019; Zhang et al., 2019; Meyerson et al., 2020). Therefore, in habitats that can support high productivity and primary metabolic rates (e.g., Atlantic Forest and Amazon rainforest), species with smaller genomes (e.g., *T. hassleriana*, *C. paludosa*, and *T. microcarpa;* Supplementary Table S1) predominate, as they can maintain higher metabolic rates and rapidly adjust their physiology to match the environmental conditions (Simonin and Roddy, 2018; Roddy et al., 2020). It is thought to occur, since the genome size restricts the minimum cell size and the maximum cell compaction densities (Simova and Herben, 2012; Roddy et al., 2020). The increased cell size represents a direct physical constraint on the number of cells that can occupy a given space and, as a result, on the distance between cell types and tissues. Furthermore, reductions in cell size, necessary to pack more veins and stomata into leaves, effectively bring actual primary productivity closer to its maximum potential (Simonin and Roddy, 2018). Thus, leaves with many small stomata and a high density of veins can maintain higher rates of gas exchange than leaves with fewer, larger stomata and larger, less numerous, veins (De Boer et al., 2012). Accordingly, variation in cell size can drive large changes in potential carbon gain (Simonin and Roddy, 2018). Regarding the effects of stress, environmental and physiological factors influence the final sizes of somatic eukaryotic cells. In this sense, the minimum size of meristematic cells and the rate of their production are strongly constrained by nuclear volume, more commonly measured as genome size (Simonin and Roddy, 2018). Meanwhile, arid habitats (e.g., Cerrado, Brazilian savanna, and Caatinga, Brazilian semi-arid region; Figure 1) are characterized by low productivity and can support species with large genomes (Roddy et al., 2020), such as *G. gynandra*, *T. spinosa*, and *T. longicarpa*, since a larger genome may be associated with greater genetic diversity (heterozygosity).

Although we hypothesize that genome size can predict the metabolic rate, its effects may be probably more nuanced, since numerous studies that have correlated the genome size with ecological factors have often produced conflicting results, and this correlation may change according to the group analyzed. Notably, studies addressing the diversity of genome size among closely related species and the association of genome size with phenotypes and ecological factors are scarce. In this light, the present study is the first to address variations in genome size within Cleomaceae, and further research is warranted to draw conclusions.

### Are *Tarenaya* Species on an Evolutionary Trajectory Toward the C_3_–C_4_ Photosynthetic Mechanism?

C_4_ photosynthesis arose from C_3_ photosynthesis through a series of events/phases (Sage, 2004, 2021; Gowik and Westhoff, 2011). Combined physiological, molecular, and morphological changes played crucial roles in the evolution of the C_4_ photosynthetic mechanism. The Cleomaceae accessions studied here were collected from warm and humid regions (Figure 1). For instance, *C. paludosa*, *T. microcarpa*, and most *T. hassleriana* accessions were collected from riverbanks in abandoned fields, except *T. hassleriana* (Joinville, SC), which was on an access road. The other species studied were collected from abandoned fields (*T*. *longicarpa* or *G. gynandra*). Similar to the species collected in the present study, some species with intermediate metabolism (e.g., *Flaveria linearis*; Holaday et al., 1984) grow in comparable environments and at disturbed sites, such as abandoned fields and roads. Thus, taking the steps essential for the development of the C_3_–C_4_ and C_4_ photosynthetic mechanisms into account (see Sage, 2004; Gowik and Westhoff, 2011) and given that the studied Cleomaceae species occur in favorable environments (Figure 1) for the development of the C_3_–C_4_ photosynthetic mechanism, our results suggest that *Tarenaya* species as well as *Cleoserrata* may be pre-conditioned to evolve the characteristics associated with carbon concentrating mechanisms. Similar trends have been observed in a previous study on *Cleome foliosa* and *Cleome africana* (Marshall et al., 2007), which exhibited increased VD and enlarged BSCs. These alterations incorporate some of the most important changes required for C_4_ photosynthesis.

Some Cleomaceae species exhibit phenotypic plasticity in leaf development and cell biology (Table 2; Marshall et al., 2007; Reeves et al., 2018), and some species have already undergone gene duplication events (Schranz and Mitchell-olds, 2006; Bergh et al., 2014). Furthermore, accessions of the same species (*T. hassleriana* and *T. longicarpa*) used in this study have a great genetic diversity, which can be observed by the different physiological, biochemical and anatomical data. This intraspecific diversity has already been observed for nine accessions of *G. gynandra* (Reeves et al., 2018) and can also be observed when comparing our accessions of *T. hassleriana*, *T. spinosa*, and *G. gynandra* with those from other continents (Table 2). However, modern phylogenetic methods allow us to analyze much more than just phylogeny as a statistical control. Specifically, they allow us to appreciate the evolutionary history of species, to better understand the patterns of biological diversity, to trace character traits over the evolutionary time, and to draw inferences regarding the evolution of these traits (Adams, 2014). As such, feature-based studies offer an important tool to better understand the ecological drivers of biological diversity. First, as observed in the present study, genome size was significantly correlated to variables essential for the C_4_ photosynthetic mechanism, such as the δ^13^C value, WUE*i*, *A*_gross_, *A*_N_, malate, aspartate, starch, proteins, VD and size of palisade parenchyma (Table 1). As demonstrated for Grasses (Wang et al., 2009) and for *G. gynandra* (Huang et al., 2021) and reported in review papers (e.g., Monson, 2003; Sage, 2004, 2021; Gowik and Westhoff, 2011), the gene duplication or whole-genome duplication is thought to facilitate the evolution of C_4_ photosynthesis from C_3_ photosynthesis (e.g., Wang et al., 2009; Bianconi et al., 2018; Tronconi et al., 2020; Huang et al., 2021). The gene duplication or whole-genome duplication creates multiple copies of the gene, allowing for modification of the copies without the original function of the transcribed protein, so to reduce selective constraint and to acquire beneficial morphological or biochemical modifications (neofunctionalization) (Monson, 2003; Huang et al., 2021). It should be noted that gene duplications occur more frequently, since it is through sexual recombination, and thus are more likely to accumulate in short-lived annuals and perennials (Sage, 2004).

Furthermore, the second step in the development of the C_4_ mechanism is characterized by increased VD (Sage, 2004; Gowik and Westhoff, 2011). The vascular system integrates with the photosynthetic tissues, and provides a flexible mechanical framework (Nelson and Dengler, 1997; Mckown and Dengler, 2010). In this way, in C_4_ plants, the vascular system takes on an additional functional role, particularly C_4_ biochemical cycling (Nelson and Dengler, 1997), due the ability to rapidly assimilate and reduce CO_2_, and survive conditions that promote the loss of carbon and energy to photorespiratory processes (Mckown and Dengler, 2010). In the present study, 15 accessions exhibited higher VD than the C_4_ species *G. gynandra*; four accessions exhibited values similar to *G. gynandra* (as verified for two *Flaveria* species C_3_ and C_4_, which presented similar VD values – Table 2); and only two accessions exhibited values lower than *G. gynandra* (Figure 3G). Of note, the VD in *G. gynandra* was equivalent to that in other accessions of the species (6–10 mm.mm^–2^) (Marshall et al., 2007; Reeves et al., 2018). However, in the remaining C_3_ accessions studied here, VD was higher than the previously reported values in the C_3_ species *C. violaceae*, *C. isomeris*, *C. hirta*, and *C. africana* as well as the C_3_-C_4_ intermediate species *Coalisina paradoxa* (Marshall et al., 2007; Table 1). These values are too higher than those observed for Brassicaceae, *Arabidopsis* (C_3_) and *Moricandia* (C_3_ and C_3_–C_4_), and Asteraceae, *Flaveria* (C_3_ and C_4_) (Table 2).

Further, differences in VD observed in the present study may also be related to increase in the mechanical integrity of leaves, which may be beneficial in windy habitats or may improve water supply to leaves in dry and hot biotopes (Sage, 2004). The minor vein density, for example, is a key determinant of leaf hydraulic capacity and photosynthetic rates, and there would be strong benefit, independently of leaf size, in an ability to vary across a wide range of environments (Sack et al., 2012). In this regard, the characteristics that may initially be an adaptation to the environment, such as the combination of shorter ID, higher VD, and larger BSC, may predispose a species to developing the C_4_ photosynthetic mechanism, as evidenced in grasses (Christin et al., 2013). Likewise, in Brassicaceae plants, VD has never been reported at the level of the C_4_ species, indicating that this anatomical attribute may be one of the major constraints to the evolution of C_4_ traits in this family (Schlüter et al., 2017). Therefore, increase in VD and enlargement of BSCs are the key alterations of foliar anatomy occurring in the C_3_ context, preceding the emergence of the C_4_ syndrome (Christin et al., 2013).

The third step in the development of the C_4_ mechanism is marked by increase in the number of organelles in BSCs, resulting in cell enlargement. Although the activation of BSCs may be a secondary effect of increase in VD (Gowik and Westhoff, 2011), some accessions such as *T. hassleriana* (Canaã, MG, and Canoinhas, SC), *T. siliculifera*, and *T*. *longicarpa* (Manaus, AM) (Supplementary Figure S8) showed BSC size comparable to *G. gynandra*. The fourth step in the evolution of the C_4_ mechanism is the appearance of glycine shuttling, a process in which the photorespiratory intermediate glycine is shuttled from the mesophyll tissue to the BSCs, where it is metabolized to CO_2_ and serine by the action of glycine decarboxylase (Sage, 2004). Plants exhibiting this trait are considered C_3_-C_4_ intermediates because they present intermediate characteristics between the C_3_ and C_4_ mechanisms.

According to a previous carbon isotope discrimination analysis, *T. siliculifera* may present a modified photosynthetic mechanism (Voznesenskaya et al., 2007). However, in the present study, the *T. siliculifera* accession exhibited a δ^13^C value equivalent to the C_3_ species, although anatomical and metabolic data indicated that it may be in transition from the C_3_ to C_3_–C_4_ metabolism. Together, these results indicate that the studied *T. siliculifera* accession warrants further investigation, at both physiological and molecular levels, to define its photosynthetic metabolism.

## Conclusion

Cleomaceae is a highly diverse family in which we can observe various types of photosynthetic metabolism, in addition to morphological differences. This diversity may be related to the evolutionary history of specific clusters, as distinct groups have different genome sizes, even within the same genus. However, this conclusion must be drawn with caution, because further evidence demonstrating that other C_4_ species in the Cleomaceae group consistently have large genomes is essential. In the present study, we also demonstrated that the C_3_-like species described here exhibit increases in VD and enlargement of BSCs, which may predispose these lineages to the development of C_4_ photosynthesis.

## Data Availability Statement

The datasets presented in this study can be found in online repositories. The names of the repository/repositories and accession number(s) can be found in the article/Supplementary Material.

## Author Contributions

DP, MV, and AN-N designed the research, analyzed the data, and wrote the manuscript with input from all the others. DP performed most of the research with the support of MV, PF, and JS. WC, PW, US, WA, RV, MS, APMW, and AN-N provided technical, logistic, and financial support. All the authors contributed to the article and approved the submitted version.

## Conflict of Interest

The authors declare that the research was conducted in the absence of any commercial or financial relationships that could be construed as a potential conflict of interest.

## Publisher’s Note

All claims expressed in this article are solely those of the authors and do not necessarily represent those of their affiliated organizations, or those of the publisher, the editors and the reviewers. Any product that may be evaluated in this article, or claim that may be made by its manufacturer, is not guaranteed or endorsed by the publisher.

## Supplementary Material

Supplementary Figure S1 Scheme of how measurements were performed in the anatomical cross-section. PP, palisade parenchyma; SP, spongy parenchyma; T, leaf thickness; BSC, bundle sheath cell width; ID, internerval distance.

Supplementary Figure S2 Molecular phylogeny of the Cleomaceae species sampled in this study. Bayesian Inference consensus tree inferred from sequences of nuclear ribosomal ITS. Numbers at nodes reflect PP. Bar: 0.02 nucleotide substitutions per site.

Supplementary Figure S3 Evolutionary regression obtained from PGLS analysis (Brownian motion) conducted with 21 species. Only the variables that presented significant *P*-value (*P* < 0.05) are shown. Physiological variables: **(A)** Ambient CO_2_ assimilation rates (A_N_), **(B)** A_gross_, **(C)** intrinsic water use efficiency (WUEi), and **(D)** respiration (R_d_). Metabolic variables: **(E)** isotopic carbon composition (δ^13^C; **^•^_/••_**), **(F)** starch content, **(G)** protein content, and **(H)** Chlorophyll *a* + *b* content. Anatomical variable: **(I)** palisade parenchyma and **(J)** density of venation. Growth parameter: **(K)** specific leaf area and **(L)** weight of 1000 seeds.

Supplementary Figure S4 Evolutionary regression obtained from PGLS analysis (Brownian motion) conducted with 21 species. Only the variables more important that presented significant *P-*value (*P* < 0.05) are shown. Relative metabolite content from GC-MS. Amino acids A-H. **(A)** α-Alanine, **(B)** glutamine, **(C)** glycine, **(D)** leucine, **(E)** methionine, **(F)** phenylalanine**, (G)** proline, **(H)** serine. Organic acids **(I–K)**. **(I)** aspartate**, (J)** γ-aminobutyric – GABA, **(K)** malate. Sugars **(L)** glucose.

Supplementary Figure S5 Gas exchange and chlorophyll *a* fluorescence parameters in Cleomaceae species. **(A)** Stomatal conductance (*g*_s_). **(B)** Intern carbon (*C*i). **(C)** Transpiration (*E*). Letters above individual box-scatter indicate significant groupings according to Tukey’s Test (*n* = 5). The median is indicated by solid lines in each box; data dispersion is represented by the interquartile range, followed by standard error and outliers. The colored bars as well as the species acronyms are related to the groups observed in Figures 1, 2.

Supplementary Figure S6 Levels of metabolites extracted from leaves of a subset of Cleomaceae species. For all metabolic analysis, leaf samples were harvested at middle of the day. **(A)** Glucose. **(B)** Fructose. **(C)** Sucrose. **(D)** Amino acids. **(E)** Chlorophyll *a*. **(F)** Chlorophyll *b*. **(G)** Chlorophyll *a* + *b*. **(H)** Chlorophyll *a*/*b.* Letters above individual box-scatter indicate significant groupings according to Tukey’s Test (*n* = 5). The median is indicated by solid lines in each box; data dispersion is represented by the interquartile range, followed by standard error and outliers. The colored bars as well as the species acronyms are related to the groups observed in Figures 1, 2.

Supplementary Figure S7 Heat map representing the changes in relative metabolite content in leaves collected at the middle of the day. Most species are 5 months old, except for TA, TM, TD, and GG which are 2 months old. The full data sets from these metabolic profiling are additionally available in Supplementary Table S3. GC-MS data was normalized by the mean of each species/metabolite. Letters indicate significant groupings according to Tukey’s Test (*n* = 5). *Indicates not significant. The colored bars as well as the species acronyms are related to the groups observed in Figures 1, 2.

Supplementary Figure S8 Natural variation of anatomical traits on leaves of a subset of Cleomaceae species. **(A)** Internerval distance. **(B)** Width bundle sheath cells. **(C)** spongy parenchyma. **(D)** Thickness. Letters above individual box-scatter indicate significant groupings according to Tukey’s Test (*n* = 5). The median is indicated by solid lines in each box; data dispersion is represented by the interquartile range, followed by standard error and outliers. The colored bars as well as the species acronyms are related to the groups observed in Figures 1, 2.

Supplementary Figure S9 Vein density in leaves of Cleomaceae species. Spots indicate presence of secretory trichomes. **(A)** THC: *T. hassleriana* (Canaã-MG). **(B)** THCS: *T. hassleriana* (Canoinhas-SC). **(C)** THD: *T. hassleriana* (Domingos Martins-ES). **(D)** THJ: *T. hassleriana* (Joinville-SC). **(E)** THP: *T. hassleriana* (Piau-MG). **(F)** THS: *T. hassleriana* (São Miguel-MG). **(G)** THV: *T. hassleriana* (Viçosa-MG). **(H)** TD: *T. diffusa* (Feira de Santana-BA. **(I)** TA: *T. aculeata* (Feira de Santana-BA). **(J)** TM: *T. microcarpa* (Belém-PA). **(K)** TSI: *T. siliculifera* (Rio Pardo-MG). **(L)** CP: *C. paludosa* (Belém-PA). **(M)** TAF: *T. longicarpa* (Afrânio-PE). **(N)** TAM: *T. longicarpa* (Manaus-AM). **(O)** TARC: *T. longicarpa* (Arcoverde-PE). **(P)** TC: *T. longicarpa* (Lavras-CE). **(Q)** TIB: *T. longicarpa* (Ibimirim-PE). **(R)** TL: *T. longicarpa* (Picos-PI). **(S)** TP: *T. parviflora* (Pombal-PB). **(T)** TR: *T. rosea* (Colatina-ES). **(U)** TS: *T. spinosa* (Teresina-PI). **(V)** GG: *G. gynandra* (Mossoró-RN). The acronyms are followed by the species name and city/state of sampling state, between parenthesis. 10x. Bars = 10 μm.

Supplementary Figure S10 Leaves cross section of Cleomaceae species. **(A)** THC: *T. hassleriana* (Canaã-MG). **(B)** THCS: *T. hassleriana* (Canoinhas-SC). **(C)** THD: *T. hassleriana* (Domingos Martins-ES). **(D)** THJ: *T. hassleriana* (Joinville-SC). **(E)** THP: *T. hassleriana* (Piau-MG). **(F)** THS: *T. hassleriana* (São Miguel-MG). **(G)** THV: *T. hassleriana* (Viçosa-MG). **(H)** TD: *T. diffusa* (Feira de Santana-BA. **(I)** TA: *T. aculeata* (Feira de Santana-BA). **(J)** TM: *T. microcarpa* (Belém-PA). **(K)** TSI: *T. siliculifera* (Rio Pardo-MG). **(L)** CP: *C. paludosa* (Belém-PA). **(M)** TAF: *T. longicarpa* (Afrânio-PE). **(N)** TAM: *T. longicarpa* (Manaus-AM). **(O)** TARC: *T. longicarpa* (Arcoverde-PE). **(P)** TC: *T. longicarpa* (Lavras-CE). **(Q)** TIB: *T. longicarpa* (Ibimirim-PE). **(R)** TL: *T. longicarpa* (Picos-PI). **(S)** TP: *T. parviflora* (Pombal-PB). **(T)** TR: *T. rosea* (Colatina-ES). **(U)** TS: *T. spinosa* (Teresina-PI). **(V)** GG: *G. gynandra* (Mossoró-RN). The acronyms are followed by the species name and city/state of sampling state, between parenthesis. 10x. Bars = 10 μm.

Supplementary Table S1 Description of the novel Brazilian species used for molecular phylogeny analyzes, and their respective collection sites (city-state abbreviation) and biomes.

Supplementary Table S2 List of ITS sequences indicating species and its respective number of identification – accession number at NCBI – used for molecular phylogeny analyses.

Supplementary Table S3 Relative metabolite content in leaves of Cleomaceae species sampled at middle of the day. Most species are 5 months old, except for TA, TM, TD, and GG, that are 2 months old. Data are presented as means ± SE. Letters indicate significant groupings according to Tukey’s Test, *n* = 5.
