# Supplementary figures and images for: New Insights Into the Evolution of C4 Photosynthesis Offered by the Tarenaya Cluster of Cleomaceae
Source: Front Plant Sci. 2022 Jan 18;12:756505. doi: 10.3389/fpls.2021.756505 (PMC8803641; doi:10.3389/fpls.2021.756505)

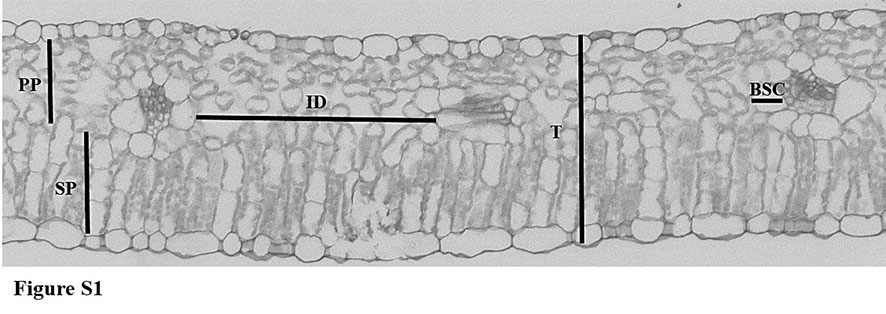

Supplement: Supplementary Figure S1 — Scheme of how measurements were performed in the anatomical cross-section. PP, palisade parenchyma; SP, spongy parenchyma; T, leaf thickness; BSC, bundle sheath cell width; ID, internerval distance. [file Image_1.JPEG]

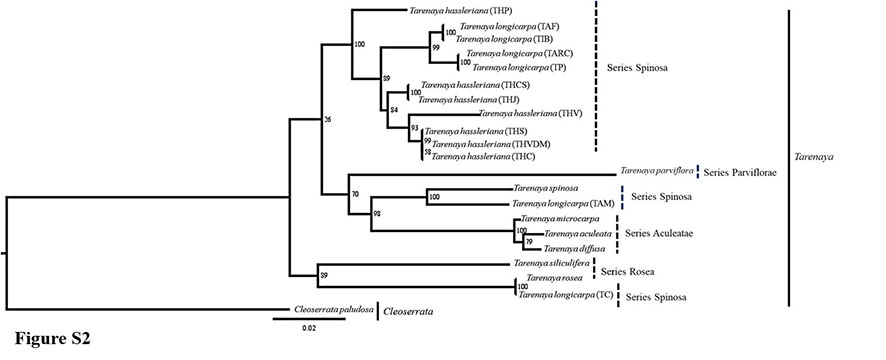

Supplement: Supplementary Figure S2 — Molecular phylogeny of the Cleomaceae species sampled in this study. Bayesian Inference consensus tree inferred from sequences of nuclear ribosomal ITS. Numbers at nodes reflect PP. Bar: 0.02 nucleotide substitutions per site. [file Image_2.JPEG]

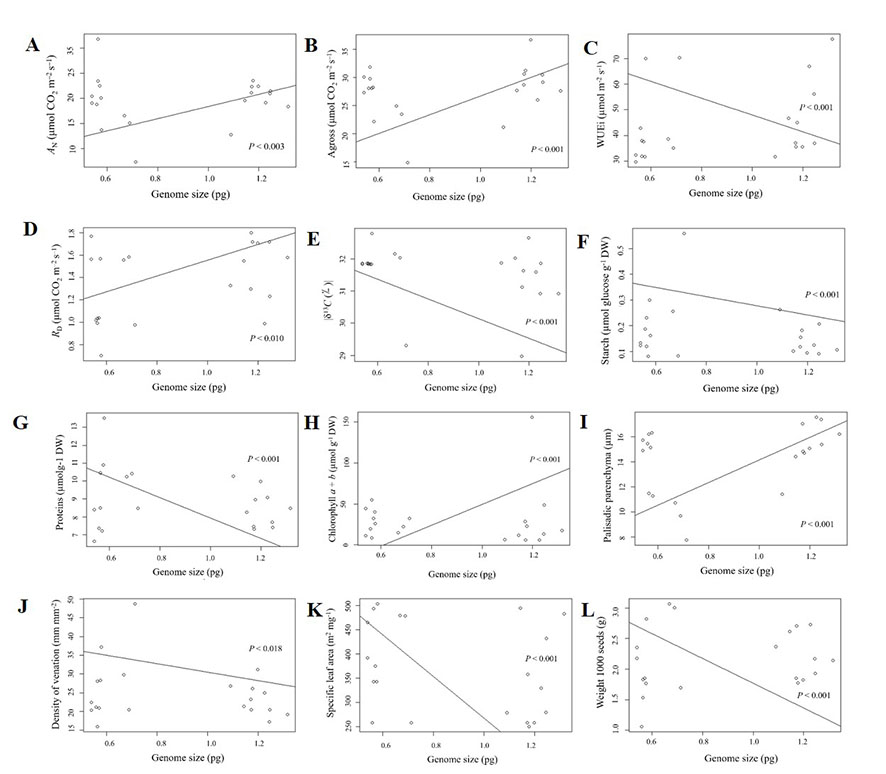

Supplement: Supplementary Figure S3 — Evolutionary regression obtained from PGLS analysis (Brownian motion) conducted with 21 species. Only the variables that presented significant P-value (P < 0.05) are shown. Physiological variables: (A) Ambient CO2 assimilation rates (AN), (B) Agross, (C) intrinsic water use efficiency (WUEi), and (D) respiration (Rd). Metabolic variables: (E) isotopic carbon composition (δ13C; •/••), (F) starch content, (G) protein content, and (H) Chlorophyll a + b content. Anatomical variable: (I) palisade parenchyma and (J) density of venation. Growth parameter: (K) specific leaf area and (L) weight of 1000 seeds. [file Image_3.JPEG]

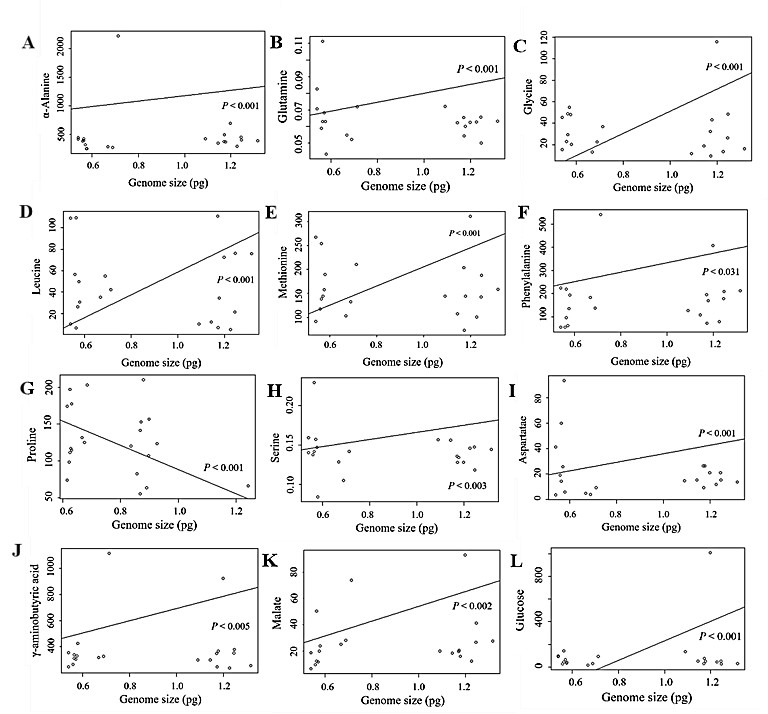

Supplement: Supplementary Figure S4 — Evolutionary regression obtained from PGLS analysis (Brownian motion) conducted with 21 species. Only the variables more important that presented significant P-value (P < 0.05) are shown. Relative metabolite content from GC-MS. Amino acids A-H. (A) α-Alanine, (B) glutamine, (C) glycine, (D) leucine, (E) methionine, (F) phenylalanine, (G) proline, (H) serine. Organic acids (I–K). (I) aspartate, (J) γ-aminobutyric – GABA, (K) malate. Sugars (L) glucose. [file Image_4.JPEG]

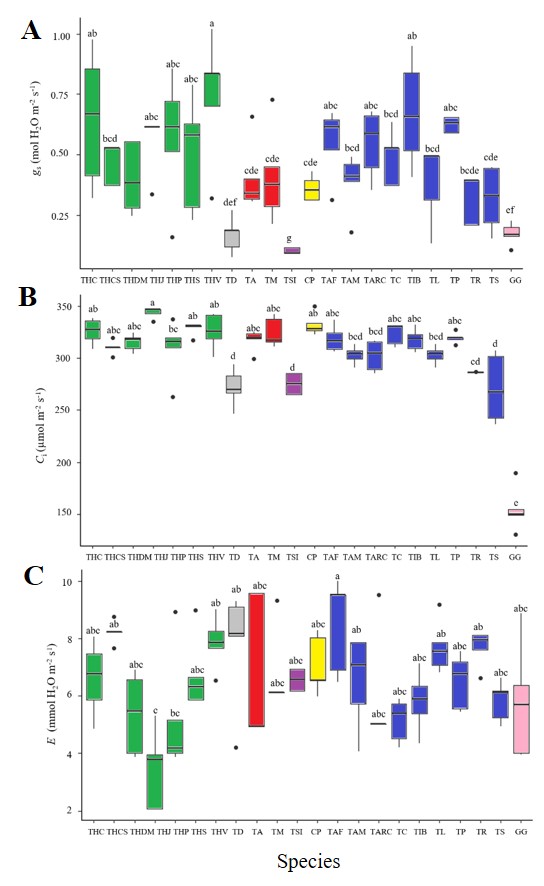

Supplement: Supplementary Figure S5 — Gas exchange and chlorophyll a fluorescence parameters in Cleomaceae species. (A) Stomatal conductance (gs). (B) Intern carbon (Ci). (C) Transpiration (E). Letters above individual box-scatter indicate significant groupings according to Tukey’s Test (n = 5). The median is indicated by solid lines in each box; data dispersion is represented by the interquartile range, followed by standard error and outliers. The colored bars as well as the species acronyms are related to the groups observed in Figures 1, 2. [file Image_5.JPEG]

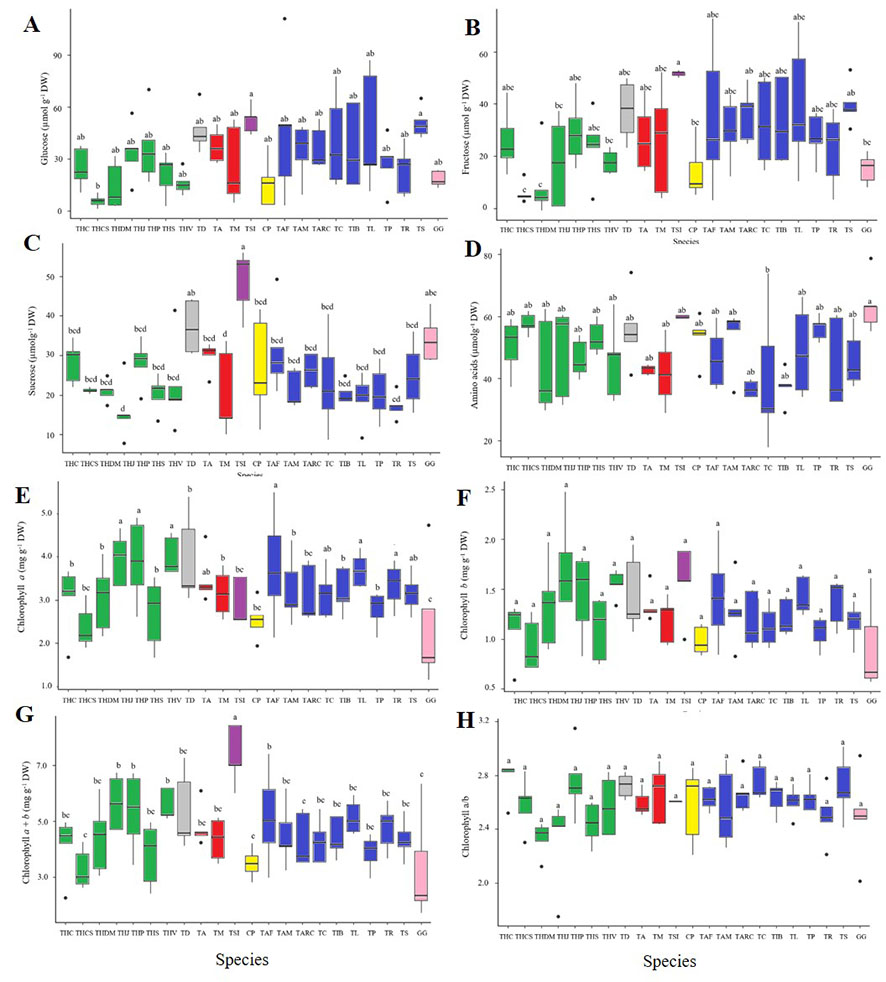

Supplement: Supplementary Figure S6 — Levels of metabolites extracted from leaves of a subset of Cleomaceae species. For all metabolic analysis, leaf samples were harvested at middle of the day. (A) Glucose. (B) Fructose. (C) Sucrose. (D) Amino acids. (E) Chlorophyll a. (F) Chlorophyll b. (G) Chlorophyll a + b. (H) Chlorophyll a/b. Letters above individual box-scatter indicate significant groupings according to Tukey’s Test (n = 5). The median is indicated by solid lines in each box; data dispersion is represented by the interquartile range, followed by standard error and outliers. The colored bars as well as the species acronyms are related to the groups observed in Figures 1, 2. [file Image_6.JPEG]

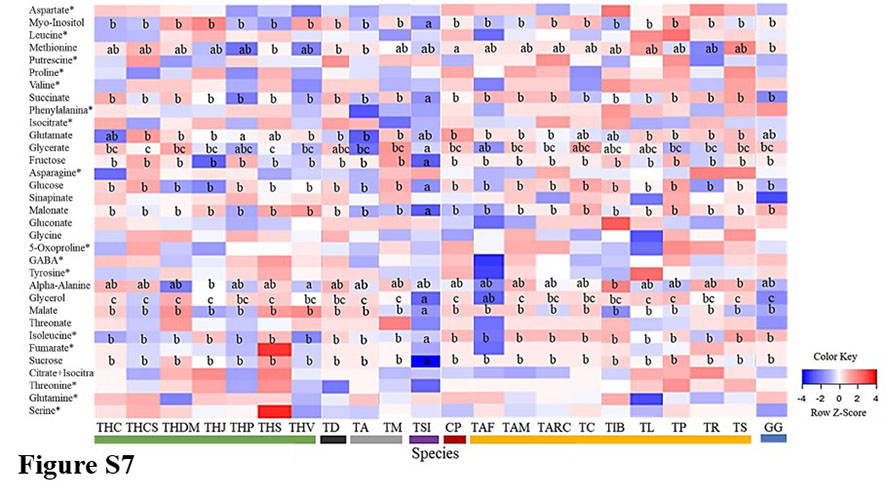

Supplement: Supplementary Figure S7 — Heat map representing the changes in relative metabolite content in leaves collected at the middle of the day. Most species are 5 months old, except for TA, TM, TD, and GG which are 2 months old. The full data sets from these metabolic profiling are additionally available in Supplementary Table S3. GC-MS data was normalized by the mean of each species/metabolite. Letters indicate significant groupings according to Tukey’s Test (n = 5). *Indicates not significant. The colored bars as well as the species acronyms are related to the groups observed in Figures 1, 2. [file Image_7.JPEG]

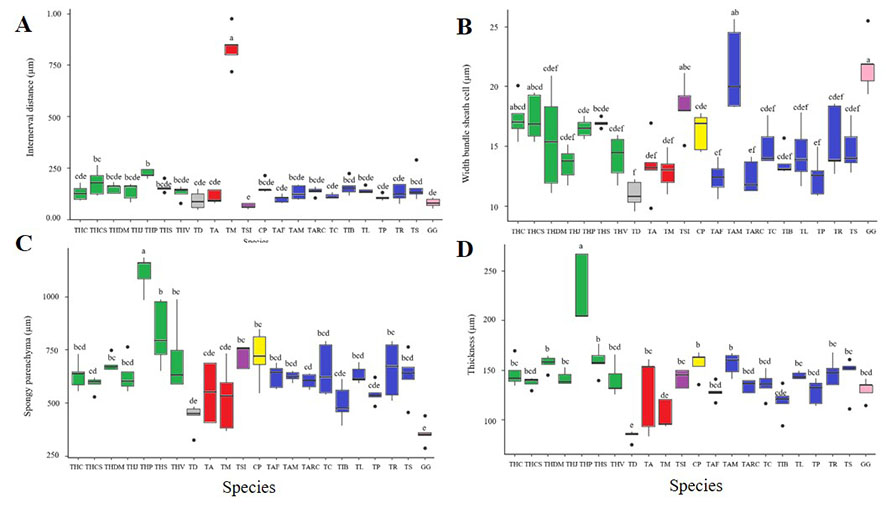

Supplement: Supplementary Figure S8 — Natural variation of anatomical traits on leaves of a subset of Cleomaceae species. (A) Internerval distance. (B) Width bundle sheath cells. (C) spongy parenchyma. (D) Thickness. Letters above individual box-scatter indicate significant groupings according to Tukey’s Test (n = 5). The median is indicated by solid lines in each box; data dispersion is represented by the interquartile range, followed by standard error and outliers. The colored bars as well as the species acronyms are related to the groups observed in Figures 1, 2. [file Image_8.JPEG]

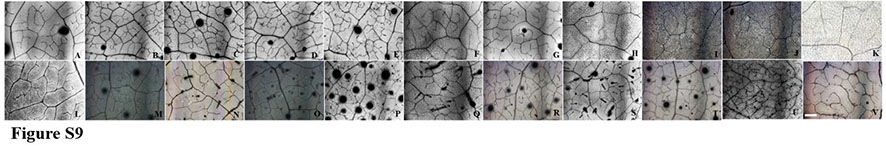

Supplement: Supplementary Figure S9 — Vein density in leaves of Cleomaceae species. Spots indicate presence of secretory trichomes. (A) THC: T. hassleriana (Canaã-MG). (B) THCS: T. hassleriana (Canoinhas-SC). (C) THD: T. hassleriana (Domingos Martins-ES). (D) THJ: T. hassleriana (Joinville-SC). (E) THP: T. hassleriana (Piau-MG). (F) THS: T. hassleriana (São Miguel-MG). (G) THV: T. hassleriana (Viçosa-MG). (H) TD: T. diffusa (Feira de Santana-BA. (I) TA: T. aculeata (Feira de Santana-BA). (J) TM: T. microcarpa (Belém-PA). (K) TSI: T. siliculifera (Rio Pardo-MG). (L) CP: C. paludosa (Belém-PA). (M) TAF: T. longicarpa (Afrânio-PE). (N) TAM: T. longicarpa (Manaus-AM). (O) TARC: T. longicarpa (Arcoverde-PE). (P) TC: T. longicarpa (Lavras-CE). (Q) TIB: T. longicarpa (Ibimirim-PE). (R) TL: T. longicarpa (Picos-PI). (S) TP: T. parviflora (Pombal-PB). (T) TR: T. rosea (Colatina-ES). (U) TS: T. spinosa (Teresina-PI). (V) GG: G. gynandra (Mossoró-RN). The acronyms are followed by the species name and city/state of sampling state, between parenthesis. 10x. Bars = 10 μm. [file Image_9.JPEG]

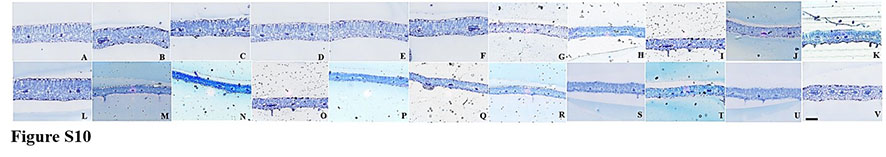

Supplement: Supplementary Figure S10 — Leaves cross section of Cleomaceae species. (A) THC: T. hassleriana (Canaã-MG). (B) THCS: T. hassleriana (Canoinhas-SC). (C) THD: T. hassleriana (Domingos Martins-ES). (D) THJ: T. hassleriana (Joinville-SC). (E) THP: T. hassleriana (Piau-MG). (F) THS: T. hassleriana (São Miguel-MG). (G) THV: T. hassleriana (Viçosa-MG). (H) TD: T. diffusa (Feira de Santana-BA. (I) TA: T. aculeata (Feira de Santana-BA). (J) TM: T. microcarpa (Belém-PA). (K) TSI: T. siliculifera (Rio Pardo-MG). (L) CP: C. paludosa (Belém-PA). (M) TAF: T. longicarpa (Afrânio-PE). (N) TAM: T. longicarpa (Manaus-AM). (O) TARC: T. longicarpa (Arcoverde-PE). (P) TC: T. longicarpa (Lavras-CE). (Q) TIB: T. longicarpa (Ibimirim-PE). (R) TL: T. longicarpa (Picos-PI). (S) TP: T. parviflora (Pombal-PB). (T) TR: T. rosea (Colatina-ES). (U) TS: T. spinosa (Teresina-PI). (V) GG: G. gynandra (Mossoró-RN). The acronyms are followed by the species name and city/state of sampling state, between parenthesis. 10x. Bars = 10 μm. [file Image_10.JPEG]
