# Supplementary material for: New Insights Into the Evolution of C4 Photosynthesis Offered by the Tarenaya Cluster of Cleomaceae
Source: Front Plant Sci. 2022 Jan 18;12:756505. doi: 10.3389/fpls.2021.756505 (PMC8803641; doi:10.3389/fpls.2021.756505)
Supplement: Supplementary Table S1 — Description of the novel Brazilian species used for molecular phylogeny analyzes, and their respective collection sites (city-state abbreviation) and biomes. [file Table_1.DOCX]

| **Species** | **ID^#^** | **Habit** | **City – State*** | **Local - Environment** | **Biome** |
| --- | --- | --- | --- | --- | --- |
| *Cleoserrata paludosa* | CP | sub-shurb | Belém – PA | marshy region | Amazon Forest |
| *Gynandropsis gynandra* | GG | herb | Mossoró – RN | crop invasive | Cerrado - Savana |
| *Tarenaya aculeata* | TA | herb | Feira de Santana – BA | open field, abandoned | Atlantic Forest |
| *T. diffusa* | TD | herb | Feira de Santana – BA | open field, abandoned | Atlantic Forest |
| *T. hassleriana* | THV | shurb | Viçosa – MG | marshy region | Atlantic Forest |
| *T. hassleriana* | THC | Shurb | Canaã – MG | open field, abandoned | Atlantic Forest |
| *T. hassleriana* | THD | Shurb | Domingos Martins – ES | roadside | Atlantic Forest |
| *T. hassleriana* | THS | Shurb | São Miguel– MG | open field, abandoned | Atlantic Forest |
| *T. hassleriana* | THSC | Shurb | Canoinhas - SC | open field, abandoned | Atlantic Forest |
| *T. hassleriana* | THP | Shurb | Piau - MG | roadside | Atlantic Forest |
| *T. hassleriana* | THJ | Shurb | Joinville – SC | roadside | Atlantic Forest |
| *T. longicarpa* | TL | Shurb | Picos – PI | open field, abandoned | Caatinga - Savana |
| *T. microcarpa* | TM | Herb | Belém – PA | marshy region | Amazon Forest |
| *T. parviflora* | TP | Shurb | Pombal – PB | marshy region | Caatinga - Savana |
| *T. rosea* | TR | Shurb | Colatina – ES | open field, abandoned | Atlantic Forest |
| *T. longicarpa* | TAM | Shurb | Manaus – AM | open field | Amazon Forest |
| *T. longicarpa* | TARC | Shurb | Arcoverde - PE | open field, abandoned | Caatinga – Savana |
| *T. longicarpa* | TIB | Shurb | Ibimirim - PE | open field, abandoned | Caatinga – Savana |
| *T. longicarpa* | TAF | Shurb | Afrânio - PE | open field, abandoned | Caatinga – Savana |
| *T. longicarpa* | TC | Shurb | Lavras do Ceará - CE | open field, abandoned | Caatinga – Savana |
| *T. spinosa* | TS | Shurb | Teresina - PI | roadside | Caatinga - Savana |
| *T. siliculifera*  ^#^Acronyms used for species identification. *States: PA – Pará; RN – Rio Grande do Norte; BA – Bahia; MG – Minas Gerais; ES – Espírito Santo; SC – Santa Catarina; PI – Piauí; PB – Paraíba; AM – Amazonas; CE – Ceará; PE – Pernambuco. | TSI | shurb | Rio Pardo - MG | altitude fields | Atlantic Forest |

**Table S1.** Description of the novel Brazilian species used for molecular phylogeny analyzes, and their respective collection sites (city-state abbreviation) and biomes.
