# Supplementary material for: New Insights Into the Evolution of C4 Photosynthesis Offered by the Tarenaya Cluster of Cleomaceae
Source: Front Plant Sci. 2022 Jan 18;12:756505. doi: 10.3389/fpls.2021.756505 (PMC8803641; doi:10.3389/fpls.2021.756505)
Supplement: Supplementary Table S2 — List of ITS sequences indicating species and its respective number of identification – accession number at NCBI – used for molecular phylogeny analyses. [file Table_2.DOCX]

| **Species** | **NCBI ID** |
| --- | --- |
| *Arabidopsis thaliana*  **Table S2.** List of ITS sequences indicating species and its respective number of identification – accession number at NCBI – used for molecular phylogeny analyses. | MG886682 |
| *Areocleome oxalidea* | HM044247 |
| *Arivela cleomoides* | KF923166 |
| *Arivela microaustralica* | HM044245 |
| *Arivela uncifera* | HM044249 |
| *Arivela viscosa* | KT588819 |
| *Brassica macrocarpa* | GQ268076 |
| *Brassica oleracea* | GQ891871 |
| *Brassica rapa* | GQ891874 |
| *Cleome Africana* | HM044222 |
| *Cleome amblyocarpa* | KF850548 |
| *Cleome Arabica* | KF805109 |
| *Cleome breyeri* | HM044258 |
| *Cleome coluteoides* | HM044224 |
| *Cleome densifolia* | KF923168 |
| *Cleome foliosa* | KF923173 |
| *Cleome houtteana* | LC144536 |
| *Cleome kalachariensis* | HM044277 |
| *Cleome khorassanica* | HM044230 |
| *Cleome linearifolia* | HM044278 |
| *Cleome luederitziana* | HM044256 |
| *Cleome melanosperma* | HM044284 |
| *Cleome monochrome* | HM044267 |
| *Cleome moritziana* | DQ455794 |
| *Cleome ornithopodioides* | KF923178 |
| *Cleome ramosissima* | KF850556 |
| *Cleome rotundifolia* | KF923182 |
| *Cleome schweinfurthii* | HM044273 |
| *Cleome stenophylla* | DQ455814 |
| *Cleome strigosa* | KF923184 |
| *Cleome stylosa* | DQ455812 |
| *Cleome torticarpa* | DQ455810 |
| *Cleome turkmena* | HM044231 |
| *Cleome violacea* | KF923185 |
| *Cleomella goodrichii* | KF217186 |
| *Cleomella hillmanii* | KF217187 |
| *Cleomella lutea* | HM044235 |
| *Cleomella obtusifolia* | KF217196 |
| *Cleomella plocasperma* | HM044234 |
| *Cleoserrata paludosa* | MF287989 |
| *Cleoserrata speciosa* | KT588831 |
| *Coalisina angustifolia* | HM044251 |
| *Coalisina diandra* | KF923169 |
| *Coalisina paradoxa* | HM044257 |
| *Corynandra aspera* | KT588829 |
| *Corynandra chelidonii* | KT588822 |
| *Corynandra feline* | KT588830 |
| *Corynandra simplicifolia* | KT588824 |
| *Dactylaena microphylla* | HM044279 |
| *Dactylaena* sp. | MN879423 |
| *Dactylaena* sp. | MN879424 |
| *Gynandropsis gynandra* | MH188313 |
| *Gynandropsis gynandra* | MH188314 |
| *Kersia kalachariensis* | HM044277 |
| *Melidiscus giganteus* | HM044283 |
| *Peritoma arborea* | KF217218 |
| *Peritoma multicaulis* | KF217228 |
| *Peritoma platycarpa* | KF217230 |
| *Peritoma serrulata* | KF217232 |
| *Physostemon lancolatum* | KF923175 |
| *Podandrogyne chiriquensis* | HM044281 |
| *Podandrogyne jamesonii* | HM044282 |
| *Podandrogyne macrophylla* | DQ455815 |
| *Polanisia dodecandra* | DQ455816 |
| *Rorida droserifolia* | HM044229 |
| *Rorida fimbriata* | HM044227 |
| *Rorida quinquenervia* | HM044228 |
| *Sieruela allamanii* | HM044270 |
| *Sieruela briquetii* | KF923165 |
| *Sieruela elegantissima* | HM044272 |
| *Sieruela hirta* | KR734333 |
| *Sieruela iberidella* | KF923174 |
| *Sieruela macrophylla* | HM044262 |
| *Sieruela maculate* | HM044263 |
| *Sieruela monophylla* | KT588821 |
| *Sieruela oxyphylla* | KF923179 |
| *Sieruela rutidosperma* | DQ455802 |
| *Sieruela schimperi* | HM044273 |
| *Sieruela stricta* | HM044259 |
| *Sieruela usambarica* | HM044274 |
| *Tarenaya aculeata* | MN879435 |
| *Tarenaya aculeata*_TA* | MT090703 |
| *Tarenaya afrospina* | HM044290 |
| *Tarenaya afrospina* | MN879436 |
| *Tarenaya afrospina* | MN879437 |
| *Tarenaya atropurpurea* | MN879438 |
| *Tarenaya bicolor* | MN906009 |
| *Tarenaya boliviensis* | DQ455785 |
| *Tarenaya chapalaensis* | DQ455800 |
| *Tarenaya curvispina* | MN879443 |
| *Tarenaya diffusa* | KF923170 |
| *Tarenaya domingensis* | KF923171 |
| *Tarenaya domingensis* | MN879444 |
| *Tarenaya domingensis* | MN879445 |
| *Tarenaya guaratinica* | MN879446 |
| *Tarenaya hassleriana* | DQ455791 |
| *Tarenaya hassleriana*_THC* | MT090700 |
| *Tarenaya hassleriana_*THCS* | MT090716 |
| *Tarenaya hassleriana*_THDM* | MT090714 |
| *Tarenaya hassleriana_*THJ* | MT090704 |
| *Tarenaya hassleriana_*THP* | MT090719 |
| *Tarenaya hassleriana_*THS* | MT090709 |
| *Tarenaya hassleriana_*THV* | MT090705 |
| *Tarenaya horrida* | MN879447 |
| *Tarenaya houtteana* | LC144536 |
| *Tarenaya lilloi* | MN879449 |
| *Tarenaya longicarpa _*TAM* | MT090707 |
| *Tarenaya longicarpa _*TARC* | MT090712 |
| *Tarenaya longicarpa _*TC* | MT090719 |
| *Tarenaya longicarpa _*TIB* | MT090713 |
| *Tarenaya longicarpa*_TAF* | MT090715 |
| *Tarenaya longicarpa_*TL* | MT090718 |
| *Tarenaya microcarpa_*TM* | MT090701 |
| *Tarenaya parviflora* | MN879456 |
| *Tarenaya parviflora_*TP* | MT090710 |
| *Tarenaya pernambucensis* | MN879458 |
| *Tarenaya pernambucensis* | MN879457 |
| *Tarenaya rosea* | MN879459 |
| *Tarenaya rosea_*TR* | MT090702 |
| *Tarenaya siliculifera* | HM044286 |
| *Tarenaya siliculifera_*TSI* | MT090717 |
| *Tarenaya spinosa* | HM044296 |
| *Tarenaya spinosa_*TS* | MT090706 |
| *Tarenaya titubans* | DQ455813 |
| *Tarenaya torticarpa* | DQ455810 |
| *Tarenaya trachycarpa* | HM044297 |
| *Tarenaya tucumanensis* | MN879465 |
| *Tarenaya tucumanensis* | DQ455811 |
| *Tarenaya virens* | MN879466 |
| *Tarenaya werdermannii*  *ITS sequences produced in this study. | MN879467 |
