# Supplementary material for: New Insights Into the Evolution of C4 Photosynthesis Offered by the Tarenaya Cluster of Cleomaceae
Source: Front Plant Sci. 2022 Jan 18;12:756505. doi: 10.3389/fpls.2021.756505 (PMC8803641; doi:10.3389/fpls.2021.756505)
Supplement: Supplementary Table S3 — Relative metabolite content in leaves of Cleomaceae species sampled at middle of the day. Most species are 5 months old, except for TA, TM, TD, and GG, that are 2 months old. Data are presented as means ± SE. Letters indicate significant groupings according to Tukey’s Test, n = 5. [file Table_3.DOCX]

|  | CP | GG | TA | TAF | TAM | TARC | TC | TD |
| --- | --- | --- | --- | --- | --- | --- | --- | --- |
| Aspartate | 213 ± 30a | 474 ± 170a | 364 ± 126a | 196 ± 31a | 169 ± 22a | 394 ± 133a | 331 ± 93a | 317 ± 72a |
| Myoinositol | 3280 ± 362b | 1193 ± 262b | 2939 ± 895b | 6014 ± 1478b | 5324 ± 671b | 4575 ± 624b | 2962 ± 461b | 7010 ± 1149b |
| Leucine | 6 ± 1.7a | 12 ± 3.3a | 14 ± 3.2a | 20 ± 8.8a | 11 ± 2.7a | 22 ± 3.8a | 12 ± 4.1a | 26 ± 7.9a |
| Methionine | 14 ± 4.4ab | 5 ± 2.1b | 4 ± 2.0b | 21 ± 5.7ab | 15 ± 6.0ab | 26 ± 6.4ab | 26 ± 10ab | 5 ± 1.5b |
| Putrescine | 13 ± 3.6a | 10 ± 2.1a | 67 ± 21.0a | 18 ± 4.2a | 33 ± 6.8a | 67 ± 18.6a | 19 ± 3.2a | 94 ± 21.5a |
| Proline | 10 ± 1.5a | 39 ± 16.4a | 15 ± 5.5a | 17 ± 1.8a | 12 ± 2.3a | 34 ± 6.9a | 30 ± 3.8a | 18 ± 2.5a |
| Valine | 144 ± 16.6a | 100 ± 20.8a | 103 ± 19.1a | 116 ± 12.1a | 107 ± 11.8a | 144 ± 14.4a | 204 ± 66a | 189 ± 39.7a |
| Succinate | 70 ± 6.6b | 77 ± 27.9b | 64 ± 21.0b | 90 ± 11.3b | 57 ± 5.2b | 67 ± 7.6b | 97 ± 22.3b | 64 ± 7.1b |
| Phenylalanine | 120 ± 15.5a | 66 ± 16.4a | 131 ± 43.9a | 210 ± 50.8a | 82 ± 10.9a | 152 ± 15.7a | 141 ± 27.9a | 114 ± 24.6a |
| Isocitrate | 14 ± 3.6a | 24 ± 7.5a | 14 ± 1.4a | 7 ± 2.3a | 8 ± 1.6a | 5 ± 2.0a | 7 ± 1.7a | 7 ± 2.7a |
| Glutamate | 642 ± 63.3b | 870 ± 103.4ab | 458 ± 78.3b | 611 ± 33.0b | 547 ± 18.3b | 791 ± 120.0b | 838 ± 201.4ab | 616 ± 81.2b |
| Glycerate | 126 ± 32.9bc | 64 ± 7.8bc | 103 ± 22.4bc | 247 ± 13.5ab | 108 ± 8.6bc | 129 ± 18.7bc | 235 ± 7.1abc | 194 ± 31.3abc |
| Fructose | 5637 ± 860.4b | 1497 ± 109.8b | 3113 ± 405.5b | 6439 ± 1025.0b | 6500 ± 1289.0b | 10386 ± 873.5b | 9093 ± 1044.7b | 5449 ± 1085.6b |
| Asparagine | 1 ± 0.1a | 10 ± 2.6a | 3 ± 0.7a | 3 ± 1.0a | 1 ± 0.2a | 7 ± 1.8a | 5 ± 1.9a | 2 ± 0.3a |
| Glucose | 4804 ± 628.8b | 1742 ± 443.2b | 3555 ± 788.8b | 6274 ± 1739.9b | 5411 ± 1055.8b | 7814 ± 652.8b | 6181 ± 111.1b | 8176 ± 1201.6b |
| Sinapinate | 10 ± 3.9a | 3 ± 1.4a | 21 ± 5.3a | 14 ± 5.3a | 5 ± 1.0a | 22 ± 10.6a | 7 ± 1.1a | 12 ± 3.1a |
| Malonate | 6 ± 0.9b | 2 ± 0.4b | 7 ± 1.6b | 11 ± 2.8b | 6 ± 0.7b | 6 ± 1.2b | 6 ± 1.4b | 7 ± 0.4b |
| Gluconate | 136 ± 53.7a | 42 ± 15.2a | 15 ± 3.6a | 42 ± 14.2a | 51 ± 21.1a | 52 ± 23.2a | 30 ± 4.7a | 39 ± 10.3a |
| Glycine | 366 ± 157.2a | 113 ± 34.0a | 435 ± 111.2a | 462 ± 124.5a | 102 ± 10.4a | 458 ± 160.9a | 800 ± 340.4a | 207 ± 43.4a |
| 5-oxoproline | 1412 ± 169.6a | 1462 ± 356.8a | 1731 ± 489.9a | 1331 ± 347.3a | 1261 ± 228.3a | 2060 ± 484.0a | 1583 ± 464.6a | 3406 ± 830.4a |
| GABA | 24 ± 3.4a | 20 ± 1.3a | 30 ± 6.0a | 20 ± 0.8a | 26 ± 2.4a | 36 ± 9.3a | 11 ± 2.7a | 46 ± 9.6a |
| Tyrosine | 11 ± 2.5a | 14 ± 2.4a | 13 ± 3.2a | 116 ± 67.8a | 18 ± 4.5a | 43 ± 8.6a | 32 ± 10.6a | 20 ± 4.1a |
| alpha-Alanine | 359 ± 29.5ab | 2271 ± 472.8ab | 358 ± 66.4ab | 259 ± 13.3ab | 511 ± 72.9ab | 804 ± 168.5ab | 908 ± 270.3ab | 581 ± 60.5ab |
| Glycerol | 299 ± 26.4c | 203 ± 42.2c | 318 ± 50.5c | 524 ± 85.8ab | 298 ± 35.3c | 365 ± 20.4bc | 347 ± 53.9bc | 425 ± 58.5bc |
| Malate | 1008 ± 245.2b | 1113 ± 399.0b | 1509 ± 69.4b | 3098 ± 1089.4b | 1370 ± 275.7b | 967 ± 220.7b | 1350 ± 213.3b | 1616 ± 238.8b |
| Threonate | 423 ± 40.5b | 323 ± 80.0b | 284 ± 98.5b | 698 ± 221.1b | 350 ± 34.6b | 373 ± 35.7b | 376 ± 57.3b | 253 ± 24.3b |
| Isoleucine | 23 ± 4.4a | 15 ± 3.6a | 45 ± 12.2a | 111 ± 47.2a | 33 ± 5.3a | 47 ± 6.4a | 114 ± 57.5a | 53 ± 9.5a |
| Fumarate | 19 ± 3.6a | 54 ± 22.2a | 25 ± 4.1a | 93 ± 43.5a | 18 ± 4.1a | 16 ± 1.2a | 19 ± 3.5a | 23 ± 1.7a |
| Sucrose | 21285 ± 1022.9b | 17052 ± 2427.5b | 20219 ± 2838.0b | 26102 ± 4254.4b | 20662 ± 2540.2b | 27077 ± 1188.0b | 24464 ± 3215.3b | 28833 ± 3095.3b |
| Citrate+Isocitrate | 483 ± 92.7a | 2775 ± 730.2a | 1489 ± 146.8a | 1535 ± 195.5a | 384 ± 94.6a | 287 ± 80.5a | 379 ± 91.5a | 1530 ± 127.2a |
| Threonine | 91 ± 7.8a | 90 ± 13.6a | 113 ± 25.4a | 156 ± 56.3a | 134 ± 30.4a | 128 ± 22.6a | 88 ± 23.2a | 224 ± 61.6a |
| Glutamine | 74 ± 23.4a | 327 ± 97.8a | 188 ± 57.3a | 63 ± 13.8a | 65 ± 13.9a | 543 ± 149.0a | 307 ± 71.3a | 181 ± 54.6a |
| Serine | 262 ± 86.2a | 194 ± 45.0a | 251 ± 61.9a | 231 ± 35.4a | 365 ± 120.7a | 975 ± 145.1a | 1023 ± 127.1a | 534 ± 113.3a |

**Table S3**. Relative metabolite content in leaves of Cleomaceae species sampled at middle of the day. Most species are five months old, except for TA, TM, TD and GG, that are two months old. Data are presented as means ± SE. Letters indicate significant groupings according to Tukey’s Test, n=5.

|  | THC | THCS | THDM | THJ | THP | THS | THV |
| --- | --- | --- | --- | --- | --- | --- | --- |
| Aspartate | 355 ± 75a | 190 ± 24a | 192 ± 53a | 139 ± 41a | 717 ± 96a | 93 ± 19a | 668 ± 92a |
| Myo-inositol | 2760 ± 576b | 2984 ± 398b | 2321 ± 401b | 2020 ± 651b | 2519 ± 342b | 3342 ± 560b | 3139 ± 493b |
| Leucine | 20 ± 7.1a | 19 ± 6.1a | 32 ± 7.0a | 8 ± 1.5a | 22 ± 2.4a | 11 ± 1.4a | 44 ± 5.7a |
| Methionine | 45 ± 8.8a | 19 ± 4.1ab | 25 ± 9.8ab | 14 ± 5.3ab | 40 ± 10.1ab | 3 ± 0.4b | 23 ± 6.0ab |
| Putrescine | 38 ± 9.9a | 4 ± 0.8a | 18 ± 7.3a | 29 ± 3.5a | 13 ± 1.3a | 3 ±1.1a | 23 ± 8.1a |
| Proline | 29 ± 6.6a | 6 ± 0.5a | 5 ± 0.8a | 6 ± 0.9a | 33 ± 5.8a | 10 ± 2.2a | 42 ± 7.4a |
| Valine | 157 ± 42.1a | 117 ± 9.2a | 143 ± 23.7a | 137 ± 4.9a | 254 ± 74.4a | 91 ± 7.3a | 267 ± 41.7a |
| Succinate | 83 ± 18.4b | 51 ± 6.2b | 54 ± 6.3b | 62 ± 6.0b | 170 ± 43.0b | 40 ± 3.8b | 103 ± 20.7b |
| Phenylalanine | 177 ± 39.1a | 98 ± 8.4a | 117 ± 20.4a | 111 ± 9.8a | 196 ± 38.5a | 74 ± 11.6a | 174 ± 7.9a |
| Isocitrate | 12 ± 2.4a | 15 ± 1.6a | 17 ± 3.3a | 8 ± 0.6a | 21 ± 5.4a | 11 ± 2.2a | 11 ± 3.2a |
| Glutamate | 613 ± 65.4ab | 547 ± 44.7b | 396 ± 63.3b | 429 ± 68.5b | 2995 ± 393.2a | 266 ± 29.5b | 2085 ± 422.0ab |
| Glycerate | 155 ± 8.7bc | 54 ± 5.1c | 61 ± 6.5bc | 96 ± 8.6bc | 138 ± 11abc | 54 ± 7.7c | 203 ± 17.3abc |
| Fructose | 6889 ± 802.2b | 1239 ± 802.2b | 3002 ± 208.9b | 7260 ± 1033.1b | 8293 ± 383.6b | 3784 ± 1760.0b | 7388 ± 1178.6b |
| Asparagine | 4 ± 0.8a | 2 ± 0.5a | 2 ± 0.7a | 2 ± 0.3a | 12 ± 1.3a | 1 ± 0.2a | 10 ± 1.0a |
| Glucose | 5513 ± 804.6b | 1152 ± 181.5b | 2439 ± 789.0b | 6046 ± 611.6b | 4182 ± 307.3b | 4583 ± 511.8b | 5133 ± 1029.5b |
| Sinapinate | 6 ± 1.1a | 15 ± 2.9a | 15 ± 3.4a | 5 ± 1.3a | 4 ± 0.9a | 6 ± 0.9a | 8 ± 1.6a |
| Malonate | 8 ± 1.5b | 8 ± 0.9b | 8 ± 1.5b | 5 ± 1.2b | 11 ± 2.0b | 10 ± 0.8b | 7 ± 1.1b |
| Gluconate | 32 ± 4.1a | 25 ± 4.6a | 63 ± 17.1a | 141 ± 9.8a | 44 ± 12.2a | 94 ± 29.0a | 96 ± 37.3a |
| Glycine | 1045 ± 393.7a | 71 ± 4.8a | 176 ± 73.6a | 257 ± 27.0a | 1219 ± 486.9a | 62 ± 6.5a | 417 ± 48.6a |
| 5-oxoproline | 3809 ± 685.2a | 627 ± 90.5a | 736 ± 247.8a | 924 ± 208.7a | 2599 ± 369.2a | 431 ± 82.3a | 2657 ± 505.2a |
| GABA | 51 ± 13.2a | 34 ± 4.9a | 36 ± 5.6a | 32 ± 6.8a | 55 ± 1.9a | 25 ± 0.7a | 65 ± 5.9a |
| Tyrosine | 48 ± 13.6a | 22 ± 6.6a | 54 ± 10.9a | 29 ± 8.1a | 48 ± 12.9a | 15 ± 3.9a | 45 ± 11.4a |
| alpha-Alanine | 758 ± 140.7ab | 288 ± 26.8b | 494 ± 243.3ab | 469 ± 17.2ab | 2252 ± 787.5a | 257 ± 51.9b | 920 ± 142.9ab |
| Glycerol | 330 ± 54.8ab | 264 ± 19.0c | 305 ± 25.1c | 312 ± 13.6c | 338 ± 24.8bc | 246 ± 12.0c | 353 ± 29.5bc |
| Malate | 549 ± 96.4b | 573 ± 127.4b | 492 ± 46.4b | 722 ± 181.6b | 1498 ± 352.1b | 647 ± 214.1b | 718 ± 191.2b |
| Threonate | 251 ± 35.9b | 381 ± 48.4b | 321 ± 35.3b | 428 ± 61.3b | 394 ± 71.9b | 416 ± 75.1b | 446 ± 47.0b |
| Isoleucine | 57 ± 20.3a | 33 ± 6.6a | 43 ± 14.4a | 23 ± 2.7a | 69 ± 24.8a | 20 ± 2.6a | 97 ± 26.8a |
| Fumarate | 19 ± 3.1a | 9 ± 1.1a | 11 ± 2.3a | 12 ± 1.0a | 50 ± 16.5a | 6 ± 1.1a | 18 ± 2.8a |
| Sucrose | 24276 ± 3792.5b | 16564 ± 1210.4b | 18553 ± 651.2b | 15076 ± 592.4b | 32268 ± 2515.7b | 17684 ± 460.7b | 27542 ± 2387.3b |
| Citrate+Isocitrate | 1312 ± 160.1a | 2081 ± 597.1a | 1105 ± 104.9a | 300 ± 42.0a | 1308 ± 134.7a | 611 ± 201.0a | 1060 ± 243.9a |
| Threonine | 130 ± 18.9a | 83 ± 11.4a | 78 ± 8.8a | 56 ± 7.4a | 157 ± 16.4a | 52 ± 3.5a | 159 ± 29.7a |
| Glutamine | 157 ± 22.4a | 25 ± 6.9a | 11 ± 2.9a | 45 ± 17.2a | 485 ± 147.5a | 9 ± 1.5a | 373 ± 88.5a |
| Serine | 933 ± 115.0a | 192 ± 67.5a | 147 ± 57.6a | 256 ± 56.4a | 2113 ± 745.2a | 39 ± 3.1a | 1170 ± 293.8a |

Continued...

|  | TIB | TL | TM | TP | TR | TS | TSI |
| --- | --- | --- | --- | --- | --- | --- | --- |
| Aspartate | 752 ± 31a | 147 ± 19a | 363 ± 43a | 224 ± 76a | 370 ± 95a | 88 ± 14a | 272 ± 17a |
| Myo-inositol | 4165 ± 846b | 3559 ± 635b | 3393 ± 1005b | 3679 ± 916b | 4062 ± 1018b | 3313 ± 323b | 3608 ± 55a |
| Leucine | 20 ± 3.6a | 8 ± 2.1a | 7.8 ± 2.0a | 5 ± 0.6a | 13 ± 2.1a | 6 ± 0.8a | 38 ± 4.2a |
| Methionine | 15 ± 4.5ab | 13 ± 3.4ab | 3 ± 0.7b | 9 ± 2.7ab | 21 ± 5.2ab | 4 ± 0.6ab | 11 ± 0.5ab |
| Putrescine | 57 ± 21.2a | 21 ± 4.2a | 4 ± 1.4a | 26 ± 8.6a | 50 ± 15.1a | 23 ± 7.2a | 22 ± 5.0a |
| Proline | 76 ± 24.8a | 23 ± 4.4a | 55 ± 18.4a | 6.9 ± 0.8a | 21 ± 6.5a | 5 ± 0.9a | 53 ± 1.5a |
| Valine | 187 ± 47.7a | 158 ± 46.1a | 132 ± 16.6a | 72 ± 8.7a | 122 ± 9.4a | 100 ± 13.3a | 250 ± 1.2a |
| Succinate | 92 ± 19.4b | 123 ± 26.6b | 64 ± 14.9b | 63 ± 10.7b | 63 ± 7.0b | 36 ± 6.3b | 384 ± 3.1a |
| Phenylalanine | 156 ± 37.1a | 123 ± 20.4a | 124 ± 30.8a | 55 ± 5.3a | 107 ± 17.2a | 63 ± 9.4a | 202 ± 20.8a |
| Isocitrate | 12 ± 4.7a | 3 ± 2.1a | 19 ± 5.3a | 11 ± 2.4a | 6 ± 1.4a | 6 ± 2.4a | 23 ± 3.4a |
| Glutamate | 1682 ± 649ab | 593 ± 73.7b | 887 ± 108.7b | 296 ± 48.9b | 598 ± 70.5b | 266 ± 32.3b | 1765 ± 78ab |
| Glycerate | 128 ± 26.2abc | 173 ± 13.7abc | 117 ± 26.4bc | 58 ± 1.7bc | 157 ± 10.0bc | 59 ± 8.1bc | 644 ± 13.1a |
| Fructose | 6774 ± 1169.6b | 7721 ± 1661.2b | 6218 ± 748.2b | 5673 ± 1255.5b | 5616 ± 476.8b | 5255 ± 183.1b | 46737 ± 623.4a |
| Asparagine | 4 ± 0.7a | 2 ± 0.6a | 4 ± 0.6a | 1 ± 0.3a | 3 ± 1.3a | 1 ± 0.4a | 10 ± 0.3a |
| Glucose | 5147 ± 1339.1b | 7876 ± 986.2b | 8288 ± 679.9b | 4027 ± 661.2b | 4109 ± 385.1b | 4235 ± 272.8b | 40997 ± 423.9a |
| Sinapinate | 5 ± 1.8a | 5 ± 1.0a | 4 ± 0.5a | 3 ± 0.2a | 4 ± 0.7a | 3 ± 0.3a | 22 ± 0.4a |
| Malonate | 8 ± 0.8b | 5 ± 1.3b | 6 ± 1.1b | 5 ± 0.8b | 8 ± 1.3b | 4 ± 0.3b | 61 ± 6.3b |
| Gluconate | 26 ± 5.5a | 29 ± 5.8a | 29 ± 8.7a | 74 ± 23.4a | 50 ± 16.7a | 42 ± 19.4a | 94 ± 6.3a |
| Glycine | 680 ± 31.6a | 843 ± 316.1a | 520 ± 234.2a | 76 ± 11.8a | 381 ± 149.0a | 72 ± 8.3a | 392 ± 58.1a |
| 5-oxoproline | 2660 ± 235.7a | 1461 ± 453.1a | 2057 ± 248.3a | 780 ± 234.4a | 1976 ± 600.9a | 719 ± 188.9a | 1730 ± 20.5a |
| GABA | 17 ± 1.8a | 33 ± 3.9a | 30 ± 4.4a | 17 ± 2.6a | 40 ± 3.6a | 16 ± 1.0a | 53 ± 0.4a |
| Tyrosine | 48 ± 20.0a | 16 ± 3.6a | 22 ± 4.3a | 9 ± 1.5a | 26 ± 7.1a | 13 ± 4.2a | 42 ± 0.2a |
| alpha-Alanine | 990 ± 342.5ab | 872 ± 223.7ab | 824 ± 81.6ab | 825 ± 333.3ab | 733 ± 187.7ab | 276 ± 35.5b | 1130 ± 27.7ab |
| Glycerol | 277 ± 42.9bc | 274 ± 13.2c | 327 ± 24.5c | 245 ± 24.7c | 351 ± 18.2bc | 236 ± 40.5c | 1316 ± 10.0a |
| Malate | 1883 ± 591.0b | 1659 ± 140.6b | 1887 ± 397.7b | 899 ± 121.1b | 1160 ± 228.5b | 828 ± 217.3b | 7838 ± 71.5a |
| Threonate | 405 ± 53.4b | 391 ± 74.7b | 272 ± 58.1b | 492 ± 60.6b | 453 ± 37.2b | 290 ± 59.5b | 2626 ± 17.8a |
| Isoleucine | 79 ± 23.7a | 80 ± 26.4a | 44 ± 11.0a | 16 ± 2.2a | 55 ± 18.6a | 17 ± 2.3a | 63 ± 0.3a |
| Fumarate | 41 ± 16.9a | 27 ± 5.6a | 27 ± 4.9a | 20 ± 6.9a | 26 ± 7.7a | 12 ± 3.6a | 89 ± 1.0a |
| Sucrose | 27351 ± 4156.5b | 22400 ± 1757.7b | 24093 ± 3808.8b | 16067 ± 2358.8b | 22356 ± 2898.6b | 16338 ± 1711.0b | 133716 ± 110.0a |
| Citrate+Isocitrate | 741 ± 127.1a | 386 ± 120.3a | 2157 ± 303.3a | 259 ± 39.8a | 298 ± 36.7a | 160 ± 43.3a | 783 ± 1.6a |
| Threonine | 179 ± 55.5a | 129 ± 48.1a | 162 ± 20.2a | 55 ± 6.7a | 137 ± 29.8a | 58 ± 9.7a | 150 ± 0.6a |
| Glutamine | 237 ± 109.8a | 422 ± 49.1a | 304 ± 49.0a | 26 ± 4.6a | 313 ± 150.0a | 22 ± 5.7a | 17 ± 0.6a |
| Serine | 687 ± 222.0a | 663 ± 59.3a | 361 ± 50.6a | 170 ± 32.7a | 379 ± 87.0a | 118 ± 49.6a | 83 ± 0.1a |

Continued...

Species analyzed in this study. THV: *T. hassleriana* (Viçosa-MG); THCS: *T. hassleriana* (Canoinhas-SC); THD: *T. hassleriana* (Domingos Martins-ES); THJ: *T. hassleriana* (Joinville-SC); THP: *T. hassleriana* (Piau-MG); THS: *T. hassleriana* (São Miguel-MG); THC: *T. hassleriana* (Canaã-MG); TA: *T. aculeata* (Feira de Santana-BA); TD: *T. diffusa* (Feira de Santana-BA); CP: *C. paludosa* (Belém-PA); TSI: *T. siliculifera* (Rio Pardo-MG); TM: *T. microcarpa* (Belém-PA); TIB: *Tarenaya* sp. (Ibimirim-PE); TAM: *Tarenaya* sp. (Manaus-AM); TAF: *Tarenaya* sp. (Afrânio-PE); TC: *Tarenaya* sp. (Lavras-CE); TARC: *Tarenaya* sp. (Arcoverde-PE); TS: *T. spinosa* (Teresina-PI); TP: *T. parviflora* (Pombal-PB); TR: *T. rosea* (Colatina-ES); TL: *T. longicarpa* (Picos-PI);); GG: *G. gynandra* (Mossoró-RN). Species name (City of Sampling-state).
